# Population genetic structure and geographical variation in *Neotricula aperta* (Gastropoda: Pomatiopsidae), the snail intermediate host of *Schistosoma mekongi* (Digenea: Schistosomatidae)

**DOI:** 10.1371/journal.pntd.0007061

**Published:** 2019-01-28

**Authors:** Stephen W. Attwood, Liang Liu, Guan-Nan Huo

**Affiliations:** 1 State Key Laboratory of Biotherapy, West China Hospital, West China Medical School, Sichuan University, Chengdu, People’s Republic of China; 2 Department of Life Sciences, The Natural History Museum, London, United Kingdom; PUCRS, BRAZIL

## Abstract

**Background:**

*Neotricula aperta* is the snail-intermediate host of the parasitic blood-fluke *Schistosoma mekongi* which causes Mekong schistosomiasis in Cambodia and the Lao PDR. Despite numerous phylogenetic studies only one DNA-sequence based population-genetic study of *N*. *aperta* had been published, and the origin, structure and persistence of *N*. *aperta* were poorly understood. Consequently, a phylogenetic and population genetic study was performed, with addition of new data to pre-existing DNA-sequences for *N*. *aperta* from remote and inaccessible habitats, including one new taxon from Laos and 505 bp of additional DNA-sequence for all sampled taxa,.

**Principal findings:**

Spatial Principal Component Analysis revealed the presence of significant spatial-genetic clustering. Genetic-distance-based clustering indicated four populations with near perfect match to *a priori* defined ecogeographical regions. Spring-dwelling taxa were found to form an ecological isolate relative to other *N*. *aperta*. The poor dispersal capabilities suggested by spatial-genetic analyses were confirmed by Bayesian inference of migration rates. Population divergence time estimation implied a mid-Miocene colonisation of the present range, with immediate and rapid radiation in each ecogeographical region. Estimated effective population sizes were large (120–310 thousand).

**Conclusions:**

The strong spatial-genetic structure confirmed the poor dispersal capabilities of *N*. *aperta*—suggesting human-mediated reintroduction of disease to controlled areas as the primary reason for control failure. The isolation of the spring-dwelling taxa and ecogeographical structure suggests adaptation of sub-populations to different habitats; the epidemiological significance of this needs investigation. The large effective population sizes indicate that the high population densities observed in surveyed habitats are also present in inaccessible areas; affording great potential for recrudescence driven by animal-reservoir transmission in remote streams. Mid-Miocene colonisation implies heterochronous evolution of these snails and associated schistosomes and suggests against coevolution of snail and parasite. Heterochronicity favours ecological factors as shapers of host-parasite specificity and greater potential for escape from schistosomiasis control through host-switching.

## Author summary

The disease Mekong schistosomiasis poses a threat to the health of about 1.5 million people living near the Mekong river and its tributaries in Cambodia and Laos. It is a water-borne parasite transmitted by direct contact with water in which freshwater snails of the species *Neotricula aperta* live. Control of the snails is an effective approach to control of the parasite; however, because many suitable habitats for *N*. *aperta* occur in remote and inaccessible areas, knowledge of *N*. *aperta* population sizes and interconnectivity is insufficient for the design of effective snail control interventions. Although much of the region is difficult to survey by conventional means, population genetics can be used to estimate population structure and total size from small samples of accessible populations. The study added to existing data-sets, to give more population samples and longer DNA-sequences, together with improved analytical approaches to provide a better overview of *N*. *aperta*. The findings suggest that *N*. *aperta* in different kinds of habitats are also genetically different, with very low levels of migration between them; this genetic clustering is greater than expected from spatial distance alone. Further work is needed to determine if these different clusters vary in ability to transmit the parasite. The overall population size estimates were very large; thus suggesting that high snail population densities observed in accessible habitats are also characteristic of inaccessible populations—parasites are therefore more likely to return after disease control by immigration from remote areas. Finally, the timing of evolutionary events for snails and parasites was found to differ; this implies that the parasite may not be as strongly restricted to one species of snail as originally thought, which has implications for avoidance of parasite control by host-switching.

## Introduction

### Distribution, historical-biogeography and medical significance of *Neotricula aperta*

Mekong schistosomiasis is a debilitating disease caused by infection with the parasitic blood-fluke *Schistosoma mekongi* Voge, Bruckner & Bruce 1978 [1]. An estimated 1.5 million people in Cambodia and Laos are at risk of infection by this schistosome [2]. The life-cycles of schistosomes require a snail-intermediate host, often species within a particular genus; however, transmission of *S*. *mekongi* is known to be restricted to only a single strain of the caenogastropod snail *Neotricula aperta* (Temcharoen 1971) (Pomatiopsidae: Triculinae) [3]. Transmission of Mekong schistosomiasis is highly focal and known only from seven foci involving the Mekong and three tributary river systems; thus the total range of the parasite is a mere 300 km section of the lower Mekong drainage between Khong Island (Lao PDR or Laos) and Kratié (Cambodia) [4]. By contrast the range of the snail-intermediate host is much greater, although still markedly discontinuous and geographically limited. Prior to 2014, *N*. *aperta* was known from 31 localities in Cambodia, Laos and Thailand, involving nine river systems of the lower Mekong basin [4]; however, the snail was not known from the Mekong river, or its tributaries, upstream of Khammouanne Province in Central Laos. In 2014 *N*. *aperta* was reported from the Mekong river at Ban Tha Kathin, Sri Chiang Mai District (Nong Khai Province, Thailand), which is over 200 km upstream of any previously recorded population. Interestingly, the snails in Nong Khai were found on rocks incorporated within concrete as part of anti-erosion defences along the river [5]. *N*. *aperta* had been previously found only in naturally-sited smooth stones in shallow areas of the rivers, or on submerged wood, and never on anthropogenic constructs [6].

The three strains of *N*. *aperta*, recognised on the basis of body size and mantle pigmentation [7], do not occur in sympatry and are remarkably limited in their distribution. The α-strain, the largest, being 3.5 mm in shell height on average [6], is found only in two ephemeral pools that form beside the main channel of the Mekong river during the dry-season at Khemmarat in Northeast Thailand. The β-strain is found only in the Mul river of Northeast Thailand, close to the Mul-Mekong confluence. The γ-strain is found at all other localities, and is the smallest (1.8 mm [6]) in shell height [8]. Genetic variation in *N*. *aperta* (expressed as DNA-sequence based phylogenies) does not track polytypy (see ‘Indications of earlier population phylogenetic studies’ below). *N*. γ-*aperta* is found only in shallow, well oxygenated, gently flowing waters, with silt-free smooth rock platforms. The snail exhibits poor dispersal capabilities and does not survive well outside its habitat (even if encased in damp mud) [9,10].

Surveillance to date indicates isolation of *N*. *aperta* to Cambodia and Laos (deployment in Thailand is limited to the border region with Laos), and it is difficult to explain the presence of only one other species of *Schistosoma* Weinland 1858 endemic to the lower-Mekong (namely *Schistosoma malayensis* Greer et al. 1988, which is transmitted by species of *Robertsiella* Davis & Greer 1980, also Triculinae, in peninsular Malaysia). Theories accounting for the distribution of *S*. *mekongi* have been based on an assumption of isochronicity and phylogenetic tracking by the parasite on the snails, with both taxa arriving in the region off the Indian craton, via Tibet, in the mid-Miocene (~18 Ma) [11]. The radiation of the snails and *Schistosoma* is described as mirroring the divergence of the main rivers of Tibet, as they cut their way through China and Southeast Asia to the sea [12]. Such a vicariance theory is at odds with more recent estimates of divergence times for these taxa. For example, a Bayesian estimate using a Yule tree model and an uncorrelated log normal relaxed clock suggested a divergence time of a mere 3.8 Ma (Pliocene) for *S*. *mekongi* and its sister taxon *Schistosoma japonicum* Katsurada 1904 which colonised China [2]. In contrast, the divergence of the snail-intermediate hosts of these parasites has been dated at 10 Ma [13]. Similarly, a divergence time of 5–9 Ma [14] estimated for *Robertsiella* and *Neotricula* Davis 1986, based on the general invertebrate clock for *cox1*, are at odds with the 2.5 Ma estimated for *S*. *malayensis* and *S*. *mekongi* using a Bayesian approach as described above [2]. Similarly difficult to reconcile with the vicariance model, is that despite a supposed 18 Ma of contact between the endemic Southeast Asian *Schistosoma* and the Triculinae, there are over 90 species of Triculinae in this region, but they transmit only four species of *Schistosoma*. The problem can be resolved by decoupling the snail and schistosome histories. It is important to note that the most recent common ancestor linking *Neotricula* and *Robertsiella* is in Hunan (China), and not in the area of Tibet [15]. The fact that *Robertsiella* shows derived character states, whereas *Neotricula* is morphologically a conserved member of the Triculinae, and *S*. *mekongi* appears to be derived from an *S*. *malayensis*-like lineage in DNA-sequence-based phylogeniesis, is also evidence for phylogenetic incongruence [2]. Further, at least five species of *Neotricula* are reported from Hunan, but only one from Cambodia and Laos, and none from Tibet, Yunnan (China) or Myanmar [16]. Consequently, an alternative phylogeography was proposed, with proto-*S*. *malayensis* and *Neotricula* entering Vietnam, from Hunan, via the Red river valley [16], which had Pliocene connections with the Yangtze [17]. Both Triculinae and members of the *S*. *malayensis* clade are proposed to have entered Southeast Asia using a Vietnam to Cambodia route, but the colonisations were independent and heterochronous. In support of this, molecular dating indicates major divergence events occurring across the known range of this snail between 4 and 6.5 Ma (in response to the final Indosinian orogeny) [16], and a radiation of *S*. *mekongi* into Cambodia and northwards to Khong Island (Lao PDR) around 1.3 Ma [2]. It is hypothesised that *S*. *malayensis-mekongi* diverged from the *Schistosoma sinensium* Bao 1958 clade (comprising mainly of species limited to rodents in China and the upper Mekong drainage) during their migration from Hunan on a part of the Sunda shelf now located under the sea off the Vietnam coast [16].

### Indications of earlier population phylogenetic studies

DNA-sequence based phylogenetics detected cryptic taxa within *N*. γ-*aperta* from Northeast Thailand; these were γ-I and γ-II, with the former clustering with Cambodian and Lao snails and the latter with snails from the Xe-Bang-Fai river in Khammouanne Province of central Laos. Consideration of the life-cycle of the snails, the larger shell height and greater within clade genetic variation of γ-II, led to the proposal that this clade was in fact comprised mainly of colonists arriving in the Thai Mekong river from tributaries in central Laos early in the dry-season, whereas γ-I comprised of a predominance of locally recruited snails [6]. Following the discovery in 2003 of 11 new populations, occurring in six river systems of Lao PDR and Cambodia [18], an effort was made to sequence DNA from individual snails in these, and previously known *N*. *aperta* populations and to thereby estimate population sizes, histories, and migration rates and routes [16]. The genetic clustering method used in this analysis was a Nested Clade Analysis on a network estimated by Statistical Parsimony [19]. The study generated partial sequences for two mitochondrial genes, *cox1* and *rrnL*; however, the *cox1* locus showed incompatibilities with the infinite sites model (then a strict requirement for the approach used to date divergence events) at many sites, and could not be united into an unambiguous network by Statistical Parsimony. Consequently, the study was based solely on the *rrnL* locus. This 2008 study found two monophyletic clades within *N*. *aperta*, a spring-dwelling form of northern Lao PDR and a more widespread larger-river dwelling form of southern Laos and Cambodia. Divergence of these clades was dated to 9.3 Ma, with further divergence into sub-clades around 5 Ma. The largest estimated population sizes were found in the Mekong river clades, and these were among the fastest growing populations (followed by those of eastern Cambodia). In keeping with the Red river hypothesis described above, gene-flow was in a predominantly South to North direction.

A more recent DNA-sequence based phylogeny was published (Limpanont et al., 2015) [5] based on one new population (previously unsampled), four new samples of previously studied populations, and data previously published (Attwood & Johnston, 2001) [20] for five Mekong river populations, one Xé Bang Fai river (Khammouanne Province, Laos) *N*. *γ-aperta* population, one population of *N*. *β-aperta*, and one of *N*. *α-aperta*. The newly sequenced population was from the Mekong river at Ban Tha Kathin and, as mentioned above, this represents the first record of *N*. *aperta* upstream of Khammouanne Province in Laos. The only clade to retain monophyly in the Limpanont et al. (2015) [5] phylogeny was *N*. *β-aperta*. In contrast, Attwood & Johnston (2001) [20] (based on a sub-set of the 2015 data-set) estimated a phylogeny in which *N*. *α-aperta* was basal, and the β-strain was also basal to a clade containing all *N*. *γ-aperta* sampled. In the Limpanont et al. (2015) [5] phylogeny, all of the newly sampled snails fell into one clade, which also included *γ*-II (Khemmarat) of the Attwood & Johnston (2001) [20] study, except for Thai *γ*-I taxa of the newly sampled snails which clustered with the Limpanont et al. (2015) [5] samples of *N*. *γ-aperta* from Khemmarat and Khong Jeum (probably Khong Chiam or Khong Jiam resort, a.k.a Ban Dan in earlier publications). The new population from Nong Kai clustered with a (Xé Bang Fai river,*γ*-II) clade also found in the Attwood & Johnston (2001) [20] study. The Limpanont et al. (2015) [5] study also recovered the ((Khong Island, *γ*-I), Kratié) clade reported in 2001. Nevertheless, the 2015 study found differently composed *γ*-I and *γ*-II clades, such as a break down of the distinction between Thai and Lao+Cambodian taxa in Limpanont et al. (2015) [5].

To address discrepancies between the phylogenetic studies of Attwood & Johnston (2001) [20] and Limpanont et al. (2015) [5], and to estimate better the population genetic parameters obtained by Attwood et al. (2008) [16], the present study used a larger number of characters (1050 bp), a modern Bayesian approach to phylogeny estimation, a more flexible combined partitioning scheme and model testing approach, with greater consideration to starting parameter values in modelling nucleotide substitution and cladogenesis, and vastly greater levels of resampling for error assessment. TMRCA estimation in Attwood et al. (2008) used approaches implemented in Genetree [21]; these assume a either simple constant size or simple exponential growth coalescent model [22] and determine likelihood of summaries of the data under these models by simulation. For example, Genetree assumes the same N_e_ and θ for both populations and requires multiple runs with one parameter fixed whilst searching for the Maximum Likelihood Estimate (MLE) of another; thus by alternatively fixing and searching for the ML within pairs of parameters joint maxima are found. Consequently, analyses may proceed with MLEs at false maxima. Simultaneous maximisation is possible but is locally inaccurate, and estimates for more than two populations were computationally impractical in 2008 and remain demanding even today. Although Genetree reports posterior distributions for TMRCAs, the approach is not conventionally Bayesian as these distributions are only computed at the MLEs of the population parameters (i.e. empirical-Bayesian [23]). The present study therefore used a true Bayesian approach, also with the capacity to incorporate a greater variety of clock models, to estimate TMRCAs, as implemented in BEAST 1.8.3 [24]. The 2008 study used MIGRATE v.0.97 [25], which assumes a stable sub-population size structure over time, to estimate gene-flow among populations. Although Bayesian, MIGRATE suffers several of the aforementioned problems cited for Genetree. The present study used a more realistic Isolation with Migration model in a Bayesian estimation of posterior probability distributions for migration parameters.

Wang et al. (2014) [26] also reported a phylogeny for *N*. *aperta*; however, they focused on sampling in the lower Mekong river around Kratié, used a short sequence from the *cox1* gene (342 bp) and a phylogeny estimated by Neighbour-Joining (NJ), which is a distance based method and therefore decouples estimation of genetic distance from estimation of the tree. Consequently, such a phylogenetic method is greatly inferior to ML (or other tree+model based estimators), unlikely to work well with short sequences, and is now reserved mainly for very big data (as it is fast). Wang et al. (2014) [26] reported a phylogeny featuring a basal taxon, which the authors described as ‘another race of *N*. *aperta* or even an independent species’. The finding of such disjunction in the pattern of genetic variation in these snails further supports the need for judicious population genetic study of this snail. In addition, to the issues described above, none of the previous studies incorporated geospatial data into the analysis—the present study was the first to incorporate such data.

### Hypotheses to be tested and methodological approaches

For reasons of clarity, from here on this account will, when referring to elements of the present study, reserve the term ‘(sub-)population’ for those clusterings found to be genetically distinguishable, validated, (sub-)populations, and use ‘taxon’ for all other samples/collection sites or suspected but unevaluated populations. The term clade will be used for populations or clusters thereof in a phylogeny. The findings of Attwood et al. (2008) [16] suggested that history, rather than ecology, might best explain the absence of *S*. *mekongi* from most of Laos; this implied that a spread of Mekong schistosomiasis into central and northern Laos (and even into Thailand) was possible. Unfortunately, the computing power and analytical approaches (e.g. a network based clustering approach) available to the 2008 study were far inferior to those available today. In addition, more realistic modelling of the *cox*1 data is now possible, enabling their use in such a study. Further, the dating techniques used in the 2008 study were rather simplistic. Consequently, the present study was performed to apply modern analytical approaches to the full data-set, i.e. including both *cox1* and *rrnL*, plus one additional taxon, representing a previously unsampled river. Unlike Attwood et al. (2008) [16], the present study focused on *N*. *γ-aperta*, as low gene-flow between the strains may violate assumptions of the analyses. The samples of the Nong Kai population newly reported in 2015 [5], were not available to this study; however, the phylogeny published in 2015 indicates that this population is derived from a major river population from Khammouanne in Laos. As the latter are well sampled in this study, the addition of samples from Nong Kai was not considered critical to addressing project aims.

The aim was to evaluate the findings of Attwood et al. (2008) [16] and to consider the colonisation of Southeast Asia by *S*. *mekongi*. Of particular significance to public health is to confirm the South to North migration of the snails, the extent of gene-flow between snails in transmission foci on the Mekong river and tributaries draining into the Mekong from Laos and eastern Cambodia, and the extent of divergence between spring-dwelling and river-dwelling snail taxa. Such questions relate to the potential for expansion of the range of *N*. *aperta* (and thereby of *S*. *mekongi*) northwards into Laos unrestricted by lack of potential habitat, the role of source populations in tributaries as sources in restoration of Mekong river snail populations involved in transmission following the annual flood (and in reintroduction of *S*. *mekongi* from elsewhere), and the potential for spring-dwelling snails to act as intermediate hosts for *S*. *mekongi* and support transmission in habitats currently assumed to be free of schistosomiasis. The findings of the study are therefore of considerable value in the design and planning of future schistosomiasis control and for risk-mapping in the Mekong region.

## Materials and methods

### Snail collection, DNA-sequencing, initial handling of data and model selection

The snails involved in the study were collected in Cambodia, Laos and Thailand, as reported earlier [16], with the addition of a new taxon in Laos sampled *de novo*. Table 1 gives details of taxa sampled, sampling dates, strain of *N*. *aperta* collected, and sample codes used. Sampling procedures were as previously reported [16]. Briefly, partial sequences of the cytochrome oxidase subunit I gene (*cox1*) and the large ribosomal-RNA gene (*rrnL*) were obtained, both loci being situated in the mitochondrial genome. In addition to those properties of mitochondrial loci (e.g. maternal pattern of inheritance, and smaller effective population size) that make them suited to population genetic studies, the use of these two loci allowed incorporation of a large pre-existing data-set covering almost all of the known range of *N*. *γ-aperta*. Full details of sequencing procedures, primers and justification for choice of loci have been published elsewhere [27]. Sites with indels (totalling 3 sites) were excluded from the analyses.

**Table 1:** Details of the *Neotricula aperta* taxa studied.

Village	River System	Code	Coordinates	Province	Country	Date
Mekong river						
Ban Khi Lek	Mid-Mekong	BKL	16.036 105.301	Ubon	Thailand	07/04/01
Ban Dan	Mid-Mekong	DAN	15.319 105.504	Ubon	Thailand	13/05/02
Mophou	Lower-Mekong	MOP	14.985 105.897	Champassac	Laos	03/04/01
Hat-Xai-Khoun	Lower-Mekong*	HXK	14.519 105.870	Champassac	Laos	03/04/01
Don Kang Loi	Lower-Mekong	DKL	14.514 105.859	Champassac	Laos	03/04/01
Na Fang	Lower-Mekong	NFL	14.462 105.861	Champassac	Laos	03/04/01
Don Nang Loi	Lower-Mekong	DNL	14.373 105.871	Champassac	Laos	03/04/01
Don Phakan	Lower-Mekong	DPK	13.926 105.984	Champassac	Laos	03/04/01
Stung-Treng	Lower-Mekong	SST	13.409 105.940	Stung-Treng	Cambodia	29/04/03
San Dan	Lower-Mekong*	KSC	12.774 105.963	Sambour	Cambodia	10/05/01
Krakor	Lower-Mekong	KRK	12.505 106.014	Kratié	Cambodia	07/05/01
Central Laos						
Kong Lor	*Nam Hinboun*	KLR	17.957 104.732	Savanakhet	Laos	26/03/04
Thakhen	*Nam Pakan*	TKN	17.686 104.695	Khamouane	Laos	27/03/04
Thathot	*Nam Yom*	TOT	17.624 105.145	Khamouane	Laos	28/03/04
Yommarat	*Nam Yom*	YOM	17.604 105.172	Khamouane	Laos	20/04/01
Mahaxai	Xe Bang Fai	MXL	17.413 105.198	Khamouane	Laos	20/04/01
**Ban Kang Vang**	**Xe Noy**	**BKV**	**17.068 105.070**	**Khamouane**	**Laos**	**30/04/11**
Southern Laos						
Don Kaeng Xai	Xe Kong	DKX	14.811 106.814	Attapeu	Laos	08/05/03
Attapeu	Xe Kong	XKG	14.785 106.842	Attapeu	Laos	08/05/03
Kong-Phine	Xe Kaman	XKM	14.771 106.839	Attapeu	Laos	08/05/03
Northeast Cambodia						
Sri Goh	Xe San*	SRG	13.614 106.375	Stung-Treng	Cambodia	24/04/03
Sadao	Xe Kong*	SDO	13.610 106.099	Stung-Treng	Cambodia	25/04/03
Krabi Chum	Xe San	RAM	13.553 106.517	Stung-Treng	Cambodia	24/04/03
Oh Gan	Sre Pok	OHG	13.490 106.875	Rattanakiri	Cambodia	26/04/04
Diloh	Sre Pok	DIL	13.477 107.008	Rattanakiri	Cambodia	25/04/04
Jua Talai	Sre Pok*	TAL	13.474 106.996	Rattanakiri	Cambodia	25/04/04
Jua Negn Dai	Sre-Pok	JND	13.458 106.877	Rattanakiri	Cambodia	26/04/04

For primary stream/spring taxa the drainage (or in cases where the stream was too small to be named, the major drainage with which they are associated) is given in italics. Taxa known to be implicated in the transmission of *Schistosoma mekongi* are indicated*. The new taxon sampled for this study is given in bold. Dates of collection are given.

DNA-sequence data used in the analyses have been deposited in the GenBank as follows:

*rrnL* EU306175–EU306335 (excluding Xe Noy taxon), deposited with earlier publication [16].
*cox1* KU999382-KU999795 (excluding Xe Noy taxon)
*rrnL* Xe Noy MF663267-MF663277
*cox1* Xe Noy MF663256-MF663266

For those analyses requiring an outgroup, the snail *Robertsiella silvicola* was chosen, as phylogenies for the Pomatiopsidae indicate that it lies at the root of the clade containing all Mekong river *Neotricula* [27]. The corresponding sequences, taken from the GenBank, were AF531550 and AF531548 (*cox1* and *rrnL*, respectively).

Using SeqTrace 0.9.0 [28], DNA Sequencing Chromatograms were converted into quality controlled sequences, and a consensus produced for each sample, from paired forward and reverse reads, so as to maximise final sequence quality. The resulting sequences were aligned using CLUSTAL 2.1 [29], alignments visualised using Aliview 1.17.1 [30] and trimmed using GNU Bash 4.3.48(1) (commands and extensions thereof) [31]. A concatenated *cox1*+*rrnL* ‘both loci’ alignment was created using pyfasta 0.5.2 [32] to select all *cox*1 sequences for which there was a corresponding *rrn*L sequence. The reading frame of the protein coding locus was determined using ExPASy Translate [33]. The apparently optimum partitioning strategy and corresponding evolutionary models were determined using PartitionFinder 1.0.1 [34], under a BIC criterion.

### Initial tests for population structure

The generation of a resistance surface is a requisite for spatial analysis of these data. Consequently, the CRAN R 3.2.3 [35] OpenStreetMap 0.3.2 package [36] was used to obtain a high resolution map of the sampling area. The water courses (excluding those know to be unsuitable habitats for *N*. *aperta*) were then expanded (to allow for anthropochory and zoochory of snails between closely adjacent drainages). A break was introduced into the Nam Theun river to simulate the barrier now posed by the Nam Theun 2 dam, which was not then incorporated into the OpenStreetMap data-set. The image was next desaturated and brightness minimised. The negated image was then imported to R as a raster array, the grey-scale information extracted and rasterised, coordinates were then reassigned to the image, which was finally pre-projected (to the original projection matching the data) to create a cost-surface that was used to express resistance to dispersal in subsequent analyses. To account for the fact that downstream migration is more likely than upstream, a NW to SE (decreasing) cost gradient was applied to the array (mirroring the predominant flow direction and current of the rivers in the region). In effect, this resistance surface was telling the analyses that dispersal between rivers was highly unlikely unless the rivers were very close together (within 3 km), and dispersal upstream (especially in fast flowing highland streams) was less likely than downstream.

Before attempting a detailed analysis of migration patterns and phylogeography, the data were interrogated for population genetic structure. First, inter-taxon F_ST_ values were estimated using the R package hierfstat 0.04–22 [37]. The data were next tested for signs of Isolation By Distance (IBD), as the presence of significant IBD may confound interpretation of spatial-genetic variation observed, and violate the assumptions of common clustering algorithms. To achieve this, a matrix of inter-taxon distances was generated using the distcalc function [38], with slight modification (to allow data input as R variables), and the resistance surface; this provides more meaningful distances in a study of freshwater snails than simple euclidean distances. The DNA-sequence data were read into R and manipulated using R packages adegenet 2.0.2 [39] and ape 3.4 [40]. A Mantel test, implemented in R package ade4 1.7–3 [41], was then used to assess the correlation between Edwards’ distances [42] and ‘river’ distances for individual snails. The test applied Monte-Carlo sampling and 5000000 replicates. Distant and differentiated populations may show a similar pattern of genetic structure to that caused by the continuous clines of classical IBD. A simple approach to distinguish the two processes is to plot both distances [43]. To avoid granularity due to binning, 2-dimensional kernel density estimation (Parzen method) was used together with the MASS package [44] and adegenet (in R) to plot local densities, so as to distinguish true IBD from differentiation of distant populations [39].

### Assessment of population structure by PCA and simple multivariate methods

A simple assessment of geographical distribution followed by multivariate methods was used to establish the presence of distinct populations in *N*. *γ-aperta*; the use of multivariate analysis provides a rapid and flexible (i.e. independent of population genetic models) approach to determining if significant deviations from panmixis occur across the range of this snail, before attempting a more involved phylogeographical analysis. In particular, the use of linked loci (mitochondrial) violates the assumptions of model based population genetic clustering approaches such as those used in STRUCTURE [45] and BAPs [46]. Similarly, such haploid loci preclude tests based on heterozygosity deviations.

A table of (binomially transformed) standardised allele frequencies was subjected to Principal Component Analysis (PCA), as implemented in adegenet. Here PCA eigenvalues represented the amount of genetic diversity accounted for by each Principal Component (PC), and a sudden decrease in eigenvalues may correspond to the point where true structure in the data gives way to noise [39]. In the present study, the first PC, and possibly also the second, were found most likely to exhibit a relevant biological signal (as judged by inspection of the corresponding scree-plot). Again kernel density estimation is used to best depict the distribution of the genotypes on the PCs. Following common practice [39], the informative PCs (in this case the first and second) were next plotted onto the geographical space, as a first step in uniting spatial and genetic variation. The plotting was implemented using ade4. Next, Moran’s I [47] test of spatial autocorrelation along the PCs was used to evaluate any spatial clustering (i.e. populations) suggested by the plots. To achieve this, spatial connectivity was defined as the resistance surface described in the previous sub-section. Moran’s test was implemented using the spdep 0.6–13 package [48] in R. As the test requires a spatial weights matrix, costs were obtained using geographical distances extracted from the river network in the resistance surface; this was achieved using R packages gdal 2.2.1 [49], maptools 0.9–2 [50], and rgeos 0.3–23 [51]. In order to assess the spatial association of each snail with its neighbours and the precision of the sampling regime, a Moran scatter plot [52] was performed; this involves plotting the standardised haplotypic data against their lagged values (i.e. the weighted average of those of their neighbouring samples) along each PC. The values in the plot correspond to standard deviations and therefore provide a useful insight into how individuals differ from one another at increasing scales. The plot was again implemented in spdep. In addition, a geographical (NW-SE) blue to red colour gradient was applied to the snail samples on the Moran plot using R’s grDevices.

### Global multivariate tests for spatial structures

The above PCA-based tests, restrict the analyses to few out of the many PCs, and therefore fail to utilise the data fully. Spatial structures in the whole data can be detected using a Mantel permutation test [53] for correlation between the genetic and geographic distance matrices. Here the test was implemented using the R package ade4. The test uses a matrix containing Euclidean distances among individual snails calculated from the scaled genetic data (as used in the PCA above), and a matrix of the corresponding spatial distances calculated from the resistance surface (i.e. effectively ‘by river’ distances). Following a suggestive result from the Mantel test, a spatial PCA (sPCA) [54] was performed to determine what proportion of the genetic variability may be spatially structured, given the data. In addition to optimising the variance of the PCs, sPCA also optimises their spatial autocorrelation through Moran’s I (i.e. the eigenvalues are composite) [55]. As with Moran’s I test, the input for the sPCA was the standardised haplotype data and the matrix of spatial proximities obtained from the resistance surface. Implementation was through adegenet.

Two randomisation tests are available in adegenet to determine which scale of structures are interpretable for the data [54]. In these tests the standardised haplotype frequency matrix used in sPCA, and list of weights derived from the resistance surface, is regressed onto Moran’s Eigenvector Maps (MEMs), and a mean *R*^*2*^ is obtained for each MEM (the highest *R*^*2*^ is taken as the test statistic and compared to a reference distribution obtained by Monte-Carlo resampling of randomly permuted frequency matrices). The global test involves decomposition of the matrix into global MEMs, and the local test into local MEMS [39]. Here 9999 permutations were used. Following these tests for interpretable structure, the spatial genetic patterns were visualised by plotting lagged global scores onto geographical space (the use of lagged scores reduces noise) [54]. The colorplot function in adegenet was used to obtain similar plots, but with a better contrasting colour range, such that similarity of colour indicates genetic affinity. Finally, Ward’s clustering method (implemented by hclust in base R) was used to assign taxa to populations inferred by minimising the energy distance between cluster groups on the first PC of the sPCA using the Ward1 algorithm [56]. The clustering was based on the differentiation of sharp changes along the PC, which define population boundaries.

### Inference of migration rates

Having obtained an estimate of population structure within *N*. *γ-aperta* by multivariate approaches, the amount and direction of gene-flow, and thereby inferred rates of migration, among these sub-populations can be estimated. To enable this, the R package phangorn 1.99.14 [57] was used to estimate a phylogeny (using NJ with a stochastic rearrangement algorithm) and then to fit HKY+G+I model parameters; these parameters were then used in the subsequent analyses where such starting values were required. As the sPCA did not suggest more than five populations, the Ward’s clustering was repeated with a permitted maximum of five sub-populations. *N*. *aperta* has been described as a metapopulation, and the analysis should therefore avoid methods such as those implemented by Migrate-N that assume stable sub-population structure over time. Nevertheless, Migrate-N 3.2.8 [58] was used in a preliminary assessment of support for combining the four populations found by Ward’s clustering into the two or three indicated by the sPCA. The Migrate-N analyses assumed a prior distribution of haplotype frequencies over populations (i.e. was Bayesian).

Several series of test runs were performed in order to select starting parameter values, settings (e.g. burn-in length, adaptive *versus* static chain heating schemes, random or UPGMA starting trees, etc) and prior distributions (e.g. on starting theta and migration rates (m_i_s), being uniform or random uniform or exponential) for the Migrate-N analyses. Initially, starting values for thetas and m_i_s were based on Fst, with later runs started using estimates of theta and m_i_ from the output of the currently best performing test-run. The relative performance of each run was assessed through Bayes-Factor (BF) tests comparing MArginal Likelihoods (MALs) determined by thermodynamic integration coupled with the use of Bezier-curves to improve approximation where a low number of heated chains is used (i.e. four here) [58]. Natural log BFs, and the guidelines of Kass and Raftery [59], in the context of theoretical expectations, were used to compare alternative runs. In addition to BFs, Effective Sample Sizes (ESSs) for estimated parameters, consistency of estimates (between replicate runs), and shape of posterior distributions, were examined for signs of good mixing and chain convergence. Runs with increasing chain length were also compared in order to optimise sampling. Consequently, analyses were performed with 20 short-chains, two replicate long-chains of 50000000 generations (taking 5000000 samples from each), a burn-in of 1000000, F_ST_ starting values and uniform priors on theta and m_i_, UPGMA starting-tree, a static heating scheme (with default values), and posterior generation by SLICE sampling. Runs with the above settings, and BFs, were then used to test alternative hypotheses regarding the number of distinct sub-populations (panmictic units) present in *N*. *aperta*.

The Migrate-N based analyses were complemented by interrogation of the data using IMa2p [60]. IMa2p might be better able to model a metapopulation as it can accommodate changes in sub-population size over time; however, this involves estimation of additional parameters and greatly increases computation time. Consequently, the analyses began with a three-population model suggested by the Migrate-N tests as best explaining the data. A per locus mutation rate of 1.3566e-04 per year was obtained from the overall meanRate of the three main BEAST runs, with the mean and 95% Highest Posterior Probability Density (HPD) passed to IMa2p as a prior. The priors on spltting time and genealogy described an island model, that is an ancestral population splitting into two in the deep past, followed by fairly constant levels of gene-flow through to the present. Test runs were initiated with a prior on this divergence of 0.8 Ma (the last major interval of river flow reversals and tectonic upheaval, the cessation of which could have segregated *N*. *aperta* populations between northern and southern Laos [27]).

Test runs, with different starting parameter values, were used to determine appropriate values to initialise the main runs. At the same time trendline plots were check for persistence of obvious trends and multiple runs, that differed only in random-number seed, were performed with the expectation that parameter estimates (and posterior probabilities) should be similar; these measures provided an indication that the Markov chains had converged in distribution. ESS values among the parameters >>50 were considered a sign of adequate mixing among parallel chains. Plots of posterior density were used to assess influence of priors (e.g. distribution greatly truncated by prior maximum, suggests prior is too low), together with runs sampling from the prior distribution alone. The full range of IMa2p’s appropriate run options (see Table 2) were evaluated, where similar posteriors were found with different settings, the simpler combination was used (e.g. fewest parameters estimated).

**Table 2:** Settings used in IMa2p runs and their meanings.

flag	setting	meaning
-hn	10	number of Markov chains run per processor (i.e. a total of 100 chains were run)
-b	20000	number of steps skipped as a burnin
-l	5.5	save genealogies every 5.5 hours
-hf	g	geometric chain-heating scheme (heated chains liberal in accepting new states)
-ha	0.999	almost linear chain heating, but β declines ~ faster for high numbered chains
-hb	0.3	lowest permitted value of β (computed following [61])
-j	7	migration rate priors are means of exponential distribution priors*
-u	1	generation time (years) (not used in models, but used to scale some output plots)
t_0_	25	upper bound for prior distribution of population splitting time (*c*.*a*. 180000 years)
-q	42.5	upper bound on population size prior (units N_e_μ) (implies N_e_ *c*.*a*. 313000)
m0>1	0.0099	mean of migration prior (exponential) distribution in direction MEK→BOL
m1>0	0.0043	mean of migration prior (exponential) distribution in direction BOL→MEK

The settings were informed by a series of test runs and Bayes Factor tests. β, the chain heating parameter. *consistent with expectation that migration rates are close to zero (exponential prior peaks at zero and has no explicit upper bound).

### Phylogenetic estimation and dating

BEAST was used to estimate a phylogeny for the individual snails sampled, and to estimate divergence times for major clades through a Bayesian approach. Bayesian phylogenetics does not assume approximate normality or large sample sizes as would general ML methods [62], and is therefore statistically superior to approaches based on unintegrated likelihoods [63]. In addition, Bayesian methods consider the posterior probability of the model (with parameters) and tree after observing the data; this is proportional to the product of the prior probability of an hypothesis and the probability of observing the dataset given the hypothesis (i.e., its likelihood), and, unlike direct ML, allows incorporation of prior information about the phylogenetic process and dates of divergence. Such incorporation of prior distributions more realistically accommodates the uncertainty associated with calibration points and estimated rates used in the analyses [64]. Although Bayesian methods have been known to erroneously converge on long-tree solutions, the present data are not partitioned and the number of parameters to be estimated is relatively low, so that the occurrence of such erroneous convergence is unlikely [65,66]. Finally, Bayesian methods are also preferred over direct ML because of their speed in terms of computing time (for analyses with an equivalent level of confidence). BEAST, which uses a Markov chain Monte Carlo (MCMC) approach to approximate the posterior probability distribution of parameters in a phylogenetic model, was chosen because of its incorporation of divergence dating and phylogenetic modelling. Molecular clock methods in BEAST generally outperform other dating approaches (e.g., non-parametric methods such as NPRS [67] or penalized likelihood methods [68]) particularly for divergences with a low time depth, as they not only allow for uncertainty in dates assigned to calibration points (through priors), but also avoid reliance on untested assumptions about the pattern of clock rate variation among lineages [69].

Initially a dated phylogeny was estimated with calibration priors on key cladogenic events. BEAST allows specification of a large number and variety of priors, starting-values and models. BFs enabled comparisons for a series of short (48 million generations) test runs used to determine optimum settings. For each test run a MAL was recorded; this MAL was the log marginal likelihood (using stepping stone sampling) obtained from pathLikelihood.delta. In brief, the tests examined the effect of changing or removing priors on substitution rates (ucld.stdev and meanRate), population size history (tree priors), offset on tree height, and date calibrations (TMRCAs and root). The purpose of such tests was to ensure that no prior was overly determinant (i.e. shaping) of the posterior distribution; this was further established by running the final analyses without the data (i.e. using the priors only), which can also reveal unpredicted problematic interactions between priors. The posterior distributions were examined using Tracer 1.5 [70]. and the run settings were only accepted if no distribution was seen to be markedly cut off by its prior or show signs of failure to converge (rising likelihood). In addition, all combinations of clock model and rate prior-distribution, implemented in BEAST, were compared (i.e. strict, fixed/random local, and Uncorrelated-Relaxed Models (URMs), with CMTMC (strict/local only), exponential*, gamma*, invgamma, Jeffry’s, lognormal, normal, and uniform prior distributions—*the only distributions tested with URM). Finally, the effect of doubling the chain length was determined for the most promising clock model and prior combinations. Indicators of likely Markov Chain convergence were trace values that reached, and then varied around, a constant log-likelihood from early in the run (just after the burnin) and thereafter, and ESSs greater than 200. Testing for the optimum length of long-chains was based around the experiences of earlier work [71] involving a similar analysis (e.g. same loci, number of sequences, etc), with adjustment for differing length of dimensions of the data. S1 Table lists the final priors, models and other run settings chosen on the basis of BFs; the MAL of this run was -8010.8614 (BF 30.43 *cf*. next best run). A plot of MAL against generation number (from 6 to 1450 million generations), for runs with all other settings as in S1 Table, was used to ensure that the likelihood had reached a stable plateau suggestive of stationarity. A chain length of 600 million was thereby found to be optimal (S1 Fig) (BF *cf*. worst and next best run respectively, 19.4583 and 9.6710). Subsequently, three such runs were performed with different random number seeds in order to confirm further convergence. No outgroup taxon was required, as the use of a relaxed clock model provided an estimate of the position of the root of the tree.

The BF based model testing suggested that substitution rates were best represented by an Uncorrelated relaxed Clock with branch-specific rates drawn from an Exponential Distribution (UCED). An exponential distribution implies that most of the branches have rates at the lower end of the range, with a few branches showing high rates. The choice of an uncorrelated model implies an episodic mode of evolution [72], which is not inconsistent with an historical biogeography described for *N*. *aperta* as dominated by cladogenesis following diastrophic events, changes in river courses and orogenies [73]. In addition, an uncorrelated clock is perhaps more realistic over the relatively short time-depth of this phylogeny and the environmental stochasticity just referenced, which are likely to overwhelm variance contributed by inherited factors. Modelling of the distribution of substitution rates across branches, for model averaging of the UCED, used an array of positive continuous parametric distributions (such as Gamma and Inverse Gaussian (IG)). Such a continuous parameterisation is considered to better accommodate rate heterogeneity; for example, the long upper tail of the IG distribution permits some taxa to have relatively high rates [74], a feature also consistent with the exponential distribution of branch-rates. It was envisaged that transition from small streams to major rivers would effect a jump in rates.

**Fig 1:**
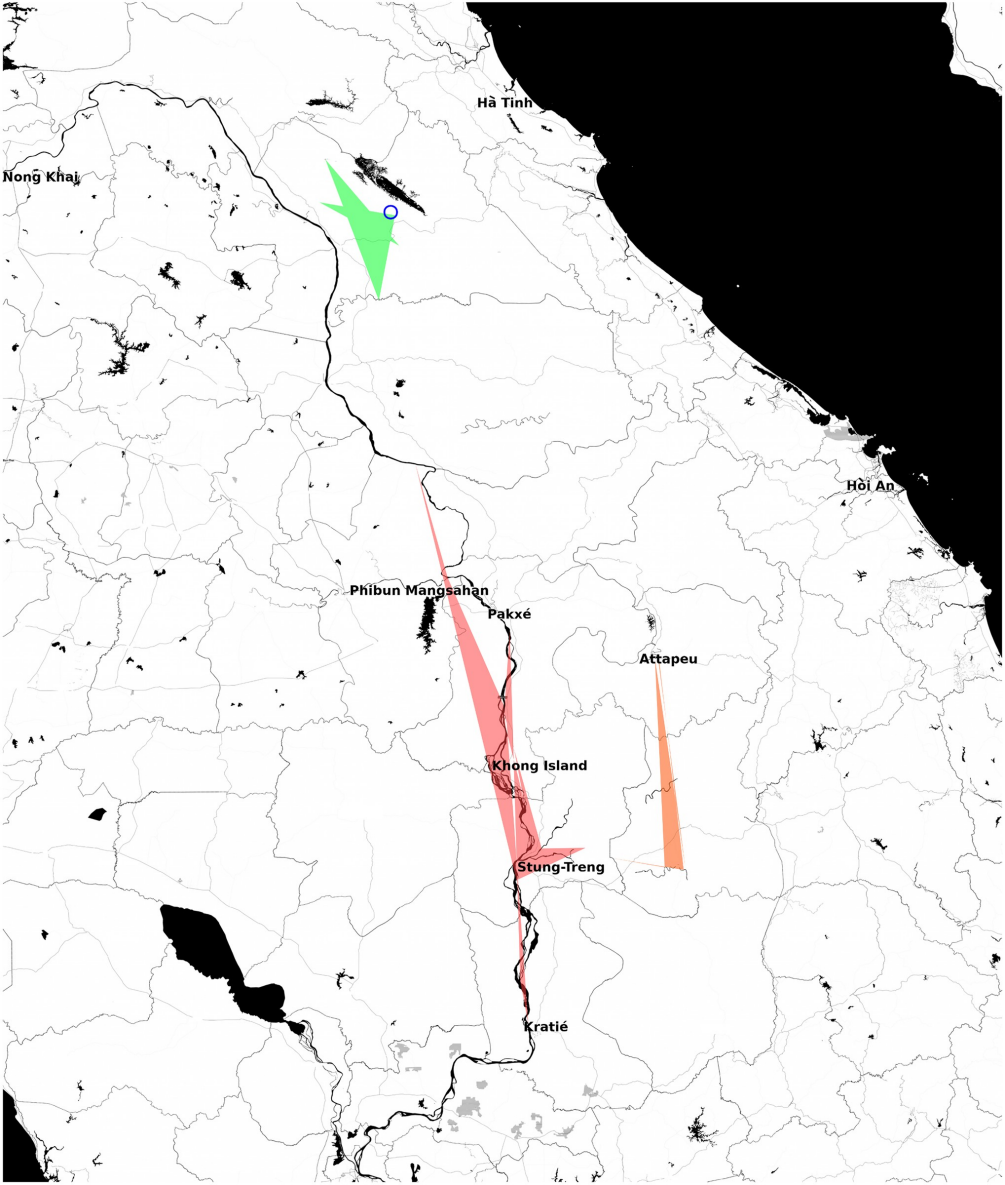
Initial reference taxa used in analyses of migration and divergence. The polygons indicate the geographical scope of sample sites in each taxon. Blue circle, CKS (TOT); green, CKR; orange, BOL; and red, MEK. Images are OpenStreetMap (OpenStreetMap contributors) data, freely available under the Open Database License. Plot was produced using the OpenStreetMap package in R.

Priors on the times of divergence events were used to guide calibration of the molecular clock, as an alternative to specifying a mean clock rate. The divergence times were MLEs of Time to Most Recent Common Ancestor (TMRCAs) taken from an earlier population phylogenetic study of *N*. *aperta* [16]. The priors were applied as normally distributed calibration dates with Standard Deviation (SD) set to achieve twice the range of the Confidence Interval (CI) of the MLE. The lower tail of these prior distributions was truncated at 0.2 Ma (megaannum or million years). Three such date priors were applied, with reference to three clades previously recognised [16] as major in the evolutionary history of *N*. *aperta*. The correspondence of these clades to sub-populations used in the migration studies of the present investigation (see Results: ‘Migration rates among sub-populations’ below) are as follows: Cambodian Eastern Rivers (CER), BOL; Northern Spring Populations (NSP), CKS+CKR; and Cambodian Lower Mekong (CLM) i.e. the southernmost Mekong river populations of Cambodia (which is MEK in part). The geographical deployment of the clades referred to in the present study are shown in Fig 1. The priors are given in S1 Table. It must be noted that these priors resulted from analysis of part of the dataset of the present study. Consequently, they are not entirely independent, but their use is equivalent to the employment of a training data sub-set to obtain parameter estimates.

The phylogeny is presented as a 50% majority rule consensus tree representing the Maximum Clade Credibility (MCC) trees of three replicate estimates, and produced using sumtrees Version 4.1.0 [75]. The MCC tree represents the topology yielding the highest product of posterior probabilities sampled for its individual constituent clades. The first 1000 trees (6 million generations) of each run were discarded as a burnin, and node support values were averaged across replicates. Phylogenetic trees were visualised using figtree 1.4.2 [76]. An array of colour ramp values was generated for geographical coordinates, running from Northeast to Southwest, using R, and the terminal branches of nexus trees, plotted using ape, were then coloured, using the plotrix package [77] in R, according to geographical location of the corresponding tip. The marginal densities of estimated parameters (e.g. TMRCAs) were examined using Tracer, and means and 95% CIs (as 95% Highest Density Interval (HDI) for the posterior distribution) are reported for the combined samples of all three replicate runs.

## Results

### Sequence data, partitions, models, and initial tests of variation

**Fig 2:**
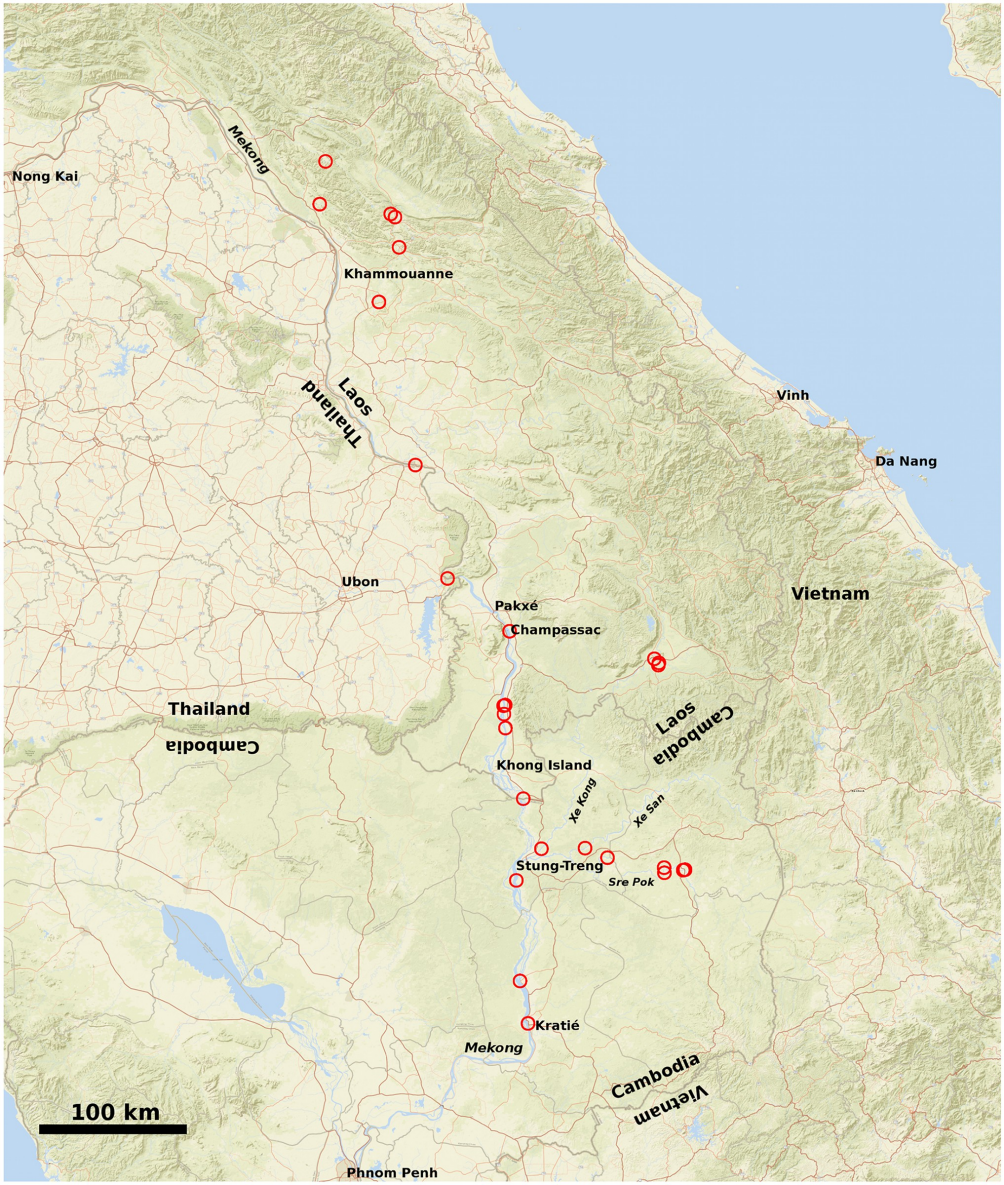
Taxa sampled. Sampling sites of *Neotricula γ-aperta* are shown as red circles. Underlying map is OpenStreetMap (OpenStreetMap contributors) data, freely available under the Open Database License. From Northwest to Southeast the area covers parts of Thailand, Laos, Cambodia, and Vietnam. River names are given in italics. Produced using the OpenStreetMap package in R.

The data-set, after editing for quality control and removal of taxa lacking quality sequence data for both loci, comprised 1050 characters (nucleotide sites or base-pairs) and 260 individuals (excluding the outgroup), representing 27 taxa of the Mekong river and nine of its tributaries in Cambodia, Laos and Thailand (Fig 2). Within the data-set, the first 545 bp corresponded to *cox1* and sites 546–1050 to the *rrnL* locus. The first character represented a third codon position. A single partition, with a HKY+I+G, model was found, by PartitionFinder, to be the simplest scenario that appeared to be least inadequate in representing the evolution of the data (BIC 17053.344).

F_ST_ values ranged from 0.024 to 0.946 (mean 0.579). The extreme F_ST_ values corresponded to DIL *versus* OHG and KRK *versus* TOT; these corresponded to almost, but not exactly, the most extreme river distances. Similarly, the Mantel test suggested high levels of IBD (*P* < 2x10^-7^) S2 Fig. A plot of the distance measures, however, showed clumping, which suggests IBD caused by well differentiated pops geographically far apart, rather than classical IBD (S3 Fig).

### Findings of the initial multivariate analysis of spatial-genetic structure

**Fig 3:**
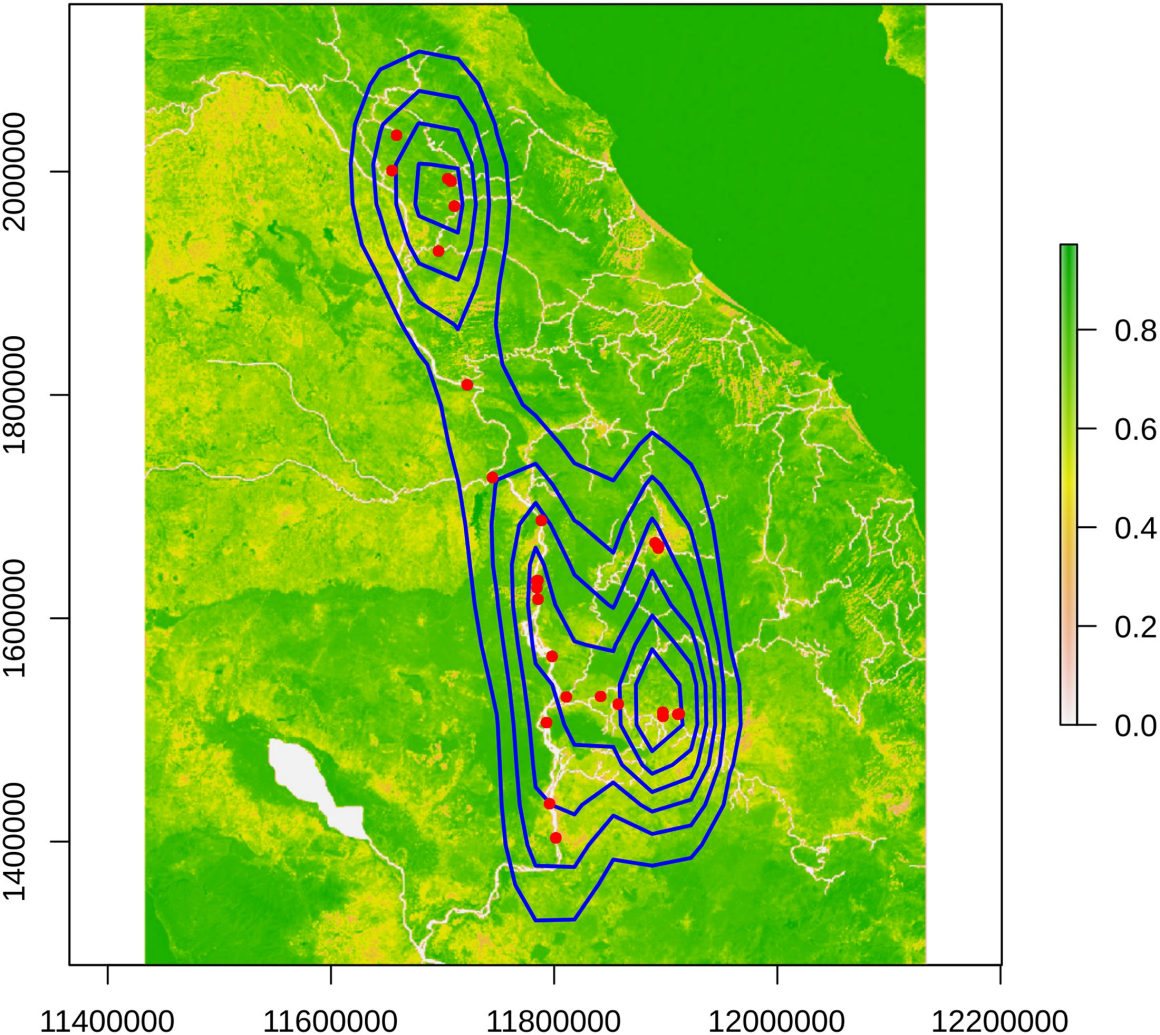
Spatial density of *Neotricula γ-aperta*. A 2-dimensional Parzen plot of the spatial density of the snail taxa on a rasterisation of the resistance surface. Plot produced using the adegenet package in R. The colour ramp to the right represents resistance (to snail dispersal) from pure green (maximal resistance) to white (minimal resistance). Underlying map based on OpenStreetMap (OpenStreetMap contributors) data, freely available under the Open Database License.

Fig 2 indicates a loose geographical clustering of the snail taxa; however, such a simple plot is a poor expression of density as it cannot account for near overlapping samples. Consequently, 2-dimensional kernel density estimation was again used to visualise better the spatial clustering. The resulting plot (Fig 3) suggested that the snail taxa were grouped into one northern and one or two southern clusters; however, none of these clusters was entirely discrete (especially the two southern groupings) and an individual-based analysis appeared more appropriate for the spatial-genetic stage of the investigation, as individuals could not be unambiguously pooled into groups.

**Fig 4:**
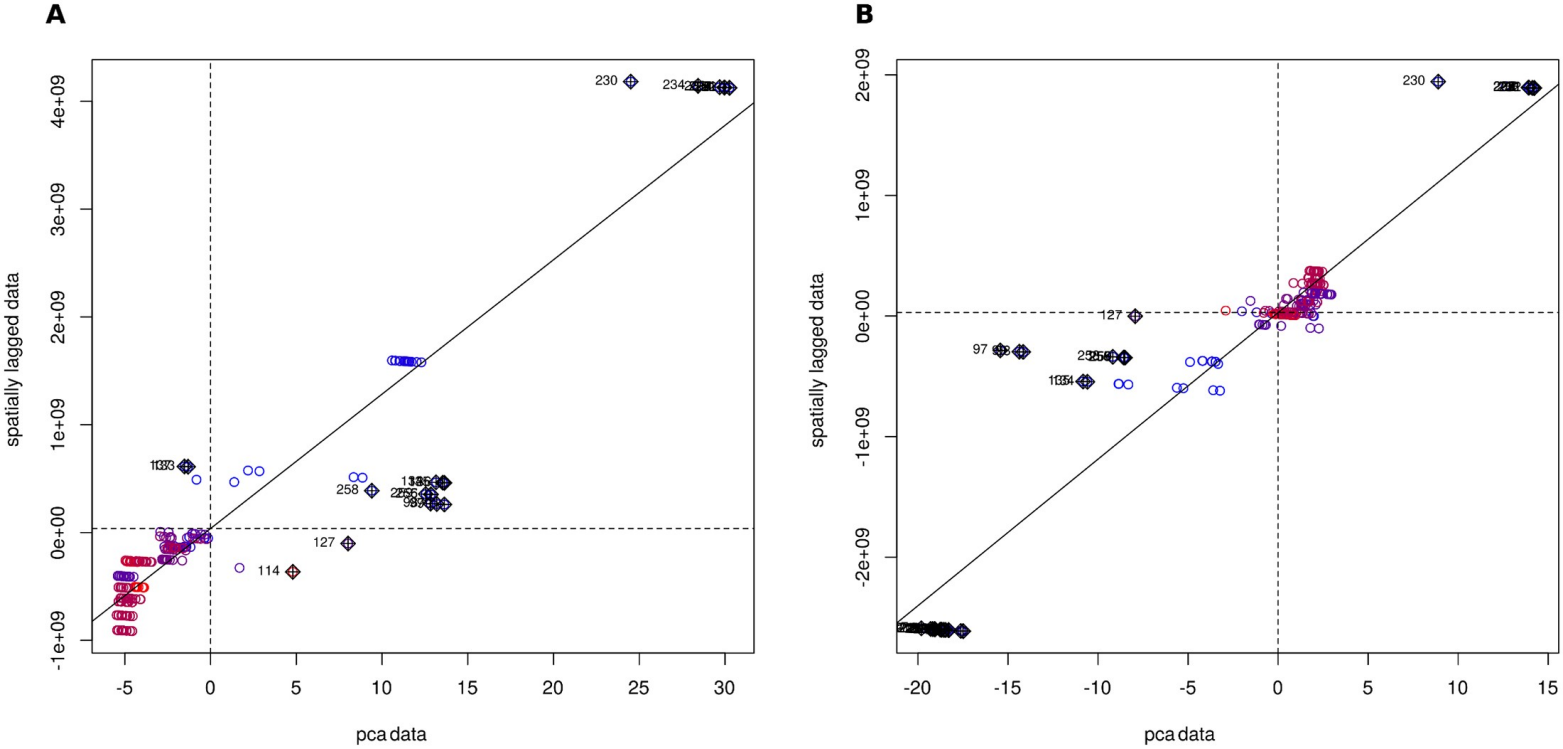
Moran scatter plots for the first two PCs of a PCA of standardised allele frequency data. A, first principal component; B, second principal component. Sampling locations are colour-gradient coded from blue to red (Northwest to Southeast), moving downstream across the range of *Neotricula γ-aperta*.

The PCA for genetic structure (genetic diversity among genotypes) indicated a number of discernible potential populations (S4A Fig). A corresponding loading plot revealed that this structure was the result of possession of many original alleles between these (S4B Fig). Moran’s I test was highly significant at both the first and second PC (S5 Fig); thus also supporting the presence of spatial-genetic clustering in the data. In both cases Moran’s I was extremely positive, suggesting that snails were likely to be surrounded by others with genotypes closely similar to their own. The Moran scatter plot for the first PC (Fig 4A) shows a concentration of Khammouane spring-dwelling snails throughout the upper-right quadrant, i.e. these snails tend to be surrounded by their close relatives and haplotype distribution is contagious (strictly, they show positive spatial autocorrelation greater than the sample mean). In contrast, snails from the center of the range show little spatial-genetic autocorrelation (i.e., they are rather panmictic), whereas those from the southeastern limit (red) are less likely to be surrounded by individuals bearing similar haplotypes, than the mean for the sample, (as they fall into the lower left quadrant). The reverse situation is seen in the Moran scatter plot for the second PC (Fig 4B); however, in this case points are clustered more around zero; this may reflect that the second PC is expected to be much less informative than the first (see scree-plot, inset to S4A Fig). Neither PC indicated negative autocorrelation (i.e. few points fell in the upper-left or lower-right quadrants), suggesting that across the range the snail haplotype distributions tend to be positively correlated, but to varying degrees. The points in both plots mostly fell close to the regression line, which suggested that there were no significant problems with the weights matrix, and that the observation (sampling) scale was sufficient for the scale of the spatial structure present.

### Global spatial patterns

The Mantel test for population structure was highly significant (S6 Fig). Consequently, spatial-genetic structure was investigated further by sPCA. Examination of the scree-plot for the sPCA indicated that the first 26 eigenvalues were positive, with only minor PCs negative (S7A Fig); this suggested overall positive spatial-genetic autocorrelation in these snails. Moran’s I ranged from -0.2 to 0.95 and the composite eigenvalue for the first PC clearly exceeded all others (S7B Fig, decomposed sPCA λ_1_). Consequently, the data contained interpretable variability and spatial structure, and only the first PC should be retained in subsequent analyses. Now that interpretable spatial-genetic structure had been detected, data regression onto MEMs was used to determine if global or local spatial structures should be interpreted. The results indicated that, whilst the test for interpretable local structure was not significant (observed value 0.009, *P* = 0.9994), that for global structure was highly significant (observed value 0.158, *P* = 0.0001).

**Fig 5:**
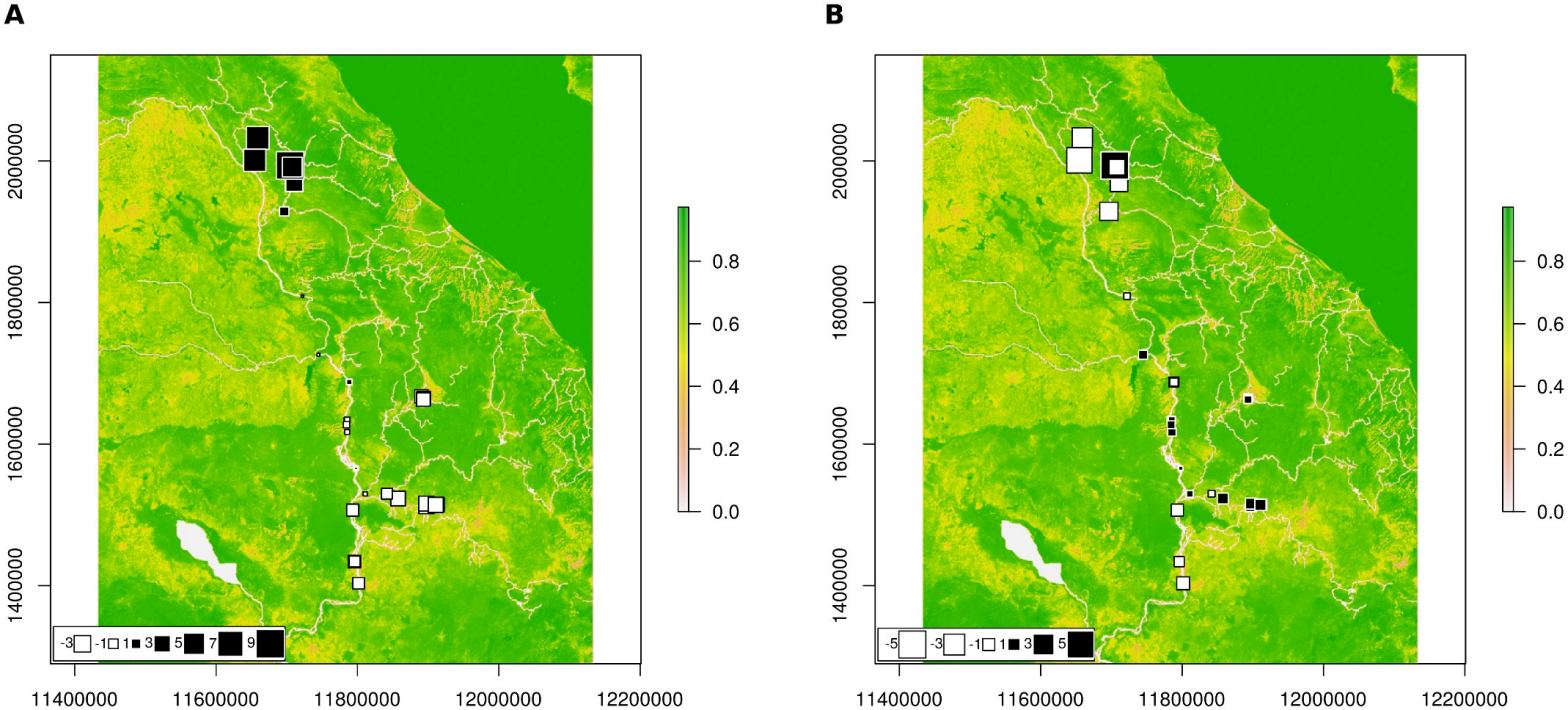
Plots of lagged global score onto geographical space for *Neotricula γ-aperta*. A, first PC; B, second PC. Large black squares represent populations that are well differentiated from those indicated by large white squares, whereas small squares indicate less differentiated populations. The colour ramp to the right represents resistance (to snail dispersal) from pure green (maximal resistance) to white (minimal resistance). Underlying map based on OpenStreetMap (OpenStreetMap contributors) data, freely available under the Open Database License.

**Fig 6:**
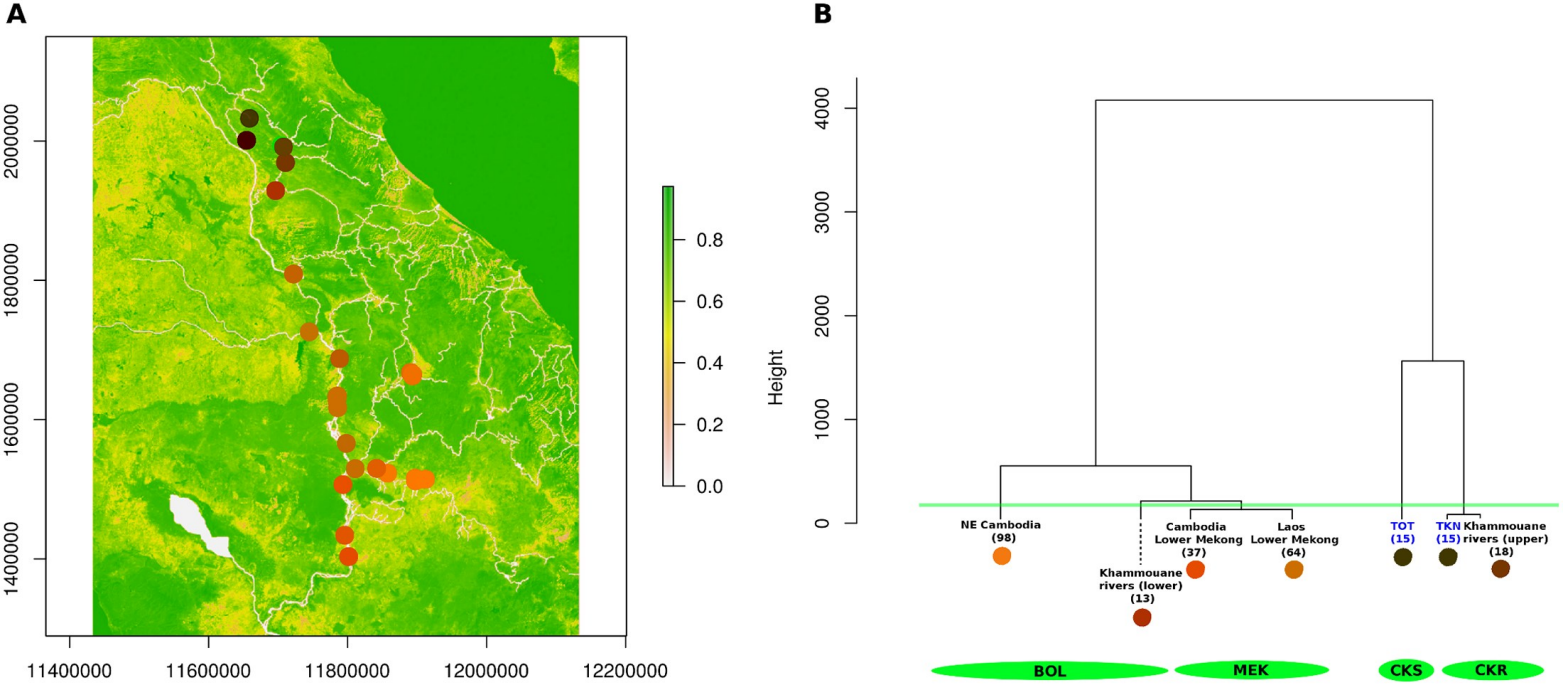
Spatial-genetic structure of *Neotricula γ-aperta*. A, colour plot of lagged global scores on geographical space; B, dendogram produced by Ward clustering. In part B, sub-population of spring-dwelling snails denoted by blue text, clade sizes (number of individuals) are given in parentheses, and the colours of the spots beneath each clade description correspond to those in the scores plot in A. The green line indicates the taxa combined to form the four sub-populations used in the migration analyses, with the sub-populations themselves shown in the green ellipses below. Underlying map in A based on OpenStreetMap (OpenStreetMap contributors) data, freely available under the Open Database License.

**Fig 7:**
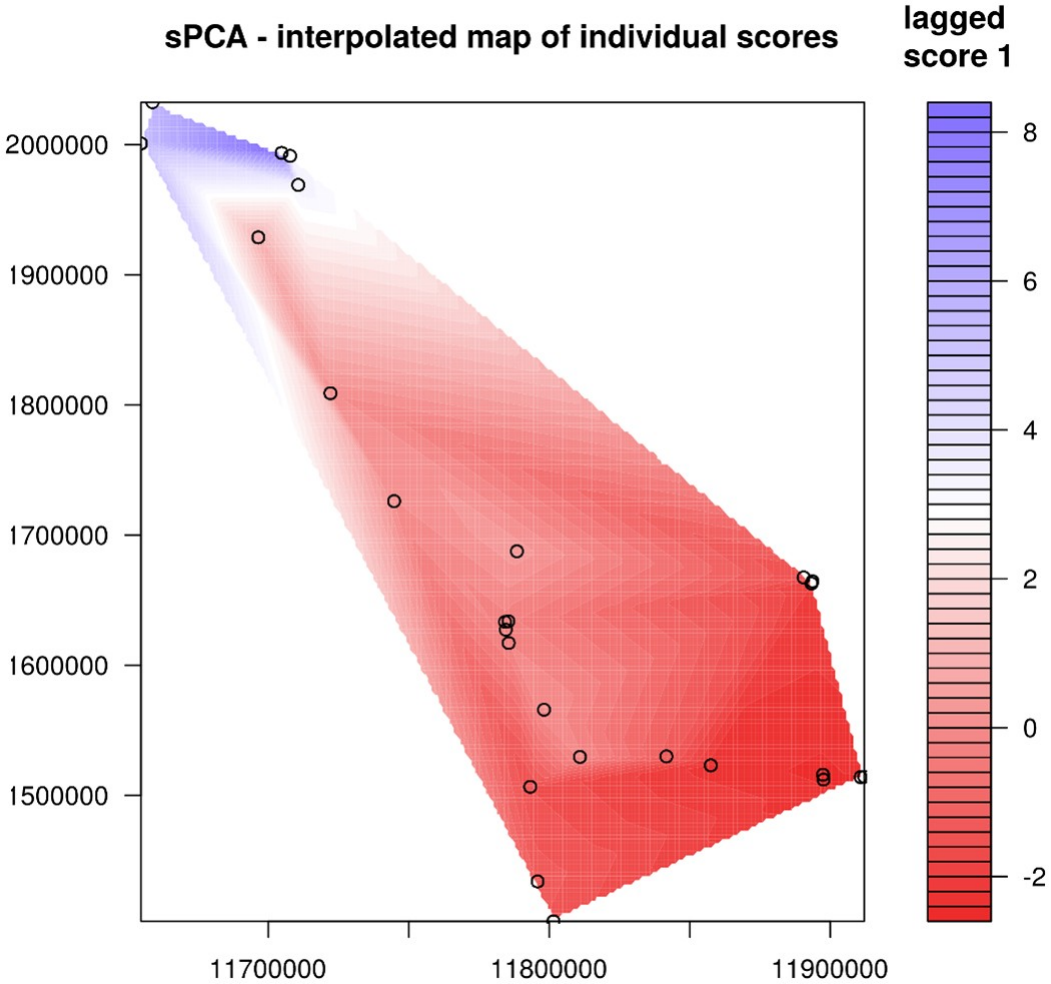
Interpolation map for *Neotricula γ-aperta*. The map was produced by interpolation of lagged scores of the principal components from the sPCA.

Plotting lagged scores onto the geographical space (Fig 5) revealed a notable North-South divide among the snail taxa; this divide is located between the Khammouane (Lao) and Ubon (Northeast Thailand) taxa in the North, and all other taxa to the South. The second PC also suggested at least a third cluster of taxa may be present in the rivers of Cambodia (Fig 5B). Fig 6A depicts genetic affinity through taxon plot colour, and indicates a general North to South cline along the Mekong river, with a moderately distinct population in the Xe Kong river and the rivers of Northeast Cambodia and a highly distinct spring-dwelling population in the Northwest. Fig 6B shows the dendogram resulting from minimum variance clustering using the first PC of the sPCA; this shows a major division in *N*. *γ-aperta*, with a clade containing all spring-dwelling taxa plus those of the upper reaches of the rivers sampled in Khammouane, and a second, larger, clade comprising all other taxa. The larger clade is divided into a sub-clade including all taxa of the rivers of northeastern Cambodia and a sub-clade containing all Mekong river taxa (including taxa of the lower reaches of the rivers in Khammouane). Finally, an interpolation map, using lagged principal scores from the sPCA, was produced (using the R package akima 0.5–12 [78]) and is provided in Fig 7 for further visualisation of genetic clines present in *N*. *γ-aperta*—the North to South cline mentioned above is seen clearly therein (i.e. the blue to red transition).

### Migration rates among sub-populations

Ward’s clustering with a maximum limit of five taxa indicated four ecogeographical populations (see Table 3 for the sub-population membership): TOT or Cammon karst small spring (CKS); Cammon karst river and large primary stream (CKR); and two flood-plain major river populations Mekong (MEK) and Bolaven (BOL). CKR comprises mainly riverine snails occurring to the South of the karst ridge in Khammouane Province (Laos)—the Cammon highland region. The Bolaven Plateau lies in southern Laos between the Mekong river on the West and the Annamite Mountain Range in the East. The upper Xe Kong river is found on this plateau. The MEK sub-population here refers to taxa of the Lao and Cambodian Mekong river itself. The four ecogeographical regions correlated well with the inferred sub-populations; however, exchange of 4 snails was required to fit the taxa precisely to the ecogeographical categories (one MOP and one KSC were moved from CKR to MEK; one DKX and one XKM were moved from MEK to BOL). These exchanges were made because the primary interest was to assess migration rates of snails among geographical regions. The relationship between the four sub-populations and those inferred by sPCA is depicted in Fig 5.

**Table 3:** Biogeographical populations indicated by Ward’s clustering.

Code	Membership
CKS	TOT
CKR	BKV, KLR, MXL, TKN, YOM
MEK	BKL, DAN, DKL, DNL, DPK, HXK, **KSC**, **KRK**, MOP, NFL, SDO, SRG, **SST**,
BOL	DIL, DKX, JND, OHG, RAM, TAL, XKG, XKM

Members of sub-population MEK-2 are given in bold (MEK-1 and -2, identified by Ward’s clustering, were merged into a single MEK taxon for subsequent analysis).

MigrateN starting parameters were as detailed in Methods: ‘Inference of migration rates’ above. Despite a Gelman’s R < 1.2, high acceptance ratios and ESS values ranging from around 70k to 1 million, replicate runs did not give consistent migration rate estimates. Consequently, the CKR and MEK populations were combined into a single population covering the karst rivers of Khammouanne immediately to the North (e.g. Xe Bang Fai drainage) and the Mekong river of Laos and Cambodia, into the northern part of which the rivers of CKR drain. The results of three replicate runs of 50 million generations each are given in Table 4 which shows considerable variance among replicates despite very similar likelihood values. The high variance of parameter estimates suggests that the data are insufficient to estimate parameters at this resolution (i.e. level of population structure); however, the particularly large variance in θ_BOL_ estimate, where sample size was not especially low (N = 100), could be partly due to further (cryptic) population structure and cross-population sampling. Greatly unequal sample sizes, e.g. TOT→WBR, would lead to unequal underestimation of θ, with θ_TOT_ being most underestimated in the example given. The observation of a very high migration rate TOT→WBR, with a relatively very low rate WBR→TOT might be due to the greater probability of dispersal, during floods, from TOT into the Nam Yom and onward downstream to MEK; however, the spring population is a phylogentically distinct ecotype, biogeographically isolated, and inflation of the θ_WBR_/θ_TOT_ ratio is an alternative explanation. Unusually high migration is also seen TOT→BOL despite these populations being isolated by highland and considerable distance (indeed, replicate 3 may have stumbled upon the true value for TOT→BOL). Overall it is likely that migration levels among all populations are between zero and ten snails per generation.

**Table 4:** Results of replicate MigrateN runs.

L	θ_TOT_	θ_CKR+MEK_	θ_BOL_	WBR→TOT	BOL→TOT	TOT→WBR	BOL→WBR	TOT→BOL	WBR→BOL
-8440.6061	0.0066	0.5692	0.4181	3.1818	1.2262	157.9036	2.2744	455.1180	5.6283
-8440.6213	0.0074	0.5112	0.3583	4.9013	2.2714	172.4338	3.5729	432.3104	8.8907
-8440.8895	0.0103	0.8211	0.0600	6.7900	0.5600	318.9000	4.7700	0.5700	0.4900
	**0.0081** **±0.002**	**0.6338** **±0.165**	**0.2788** **±0.192**	**4.9577** **±1.805**	**1.3530** **±0.863**	**216.4125** **±89.054**	**3.5391** **±1.248**	**295.9995** **±256.103**	**5.0030** **±4.235**

Migration rates as θ*m*/μ, where *m* is immigration rate (i.e. an estimate of number of immigrants per generation). L, log(Prob(Data|Model)) approximated as Bezier-curve thermodynamic marginal-likelihood; WBR, West Basin Rivers (CKR+MEK). Values in bold are means ± standard deviations.

Although perhaps unable to reliably estimate all parameters of these gene-flow models, Migrate-N was used in model testing with respect to number of distinct sub-populations present. Table 5 shows the results of the testing. The model with greatest posterior probability was that with a combined CKR+MEK population and, as the log Bayes Factor with the next ranked model was -11.0098 (which is < -2 [59]), the suggestion is that this simpler model is a better fit to the data than the original four population model.

**Table 5:** Metapopulation hypothesis testing using Migrate-N.

Model	Rank	LBF	Pr(H)
TOT, (CKR+MEK), BOL	1	NA	0.99998346
Null hypothesis	2	-11.0098	1.6539E-05
TOT, CKR, (MEK+BOL)	3	-24.5580	2.1606E-11
(TOT+CKR), MEK, BOL	4	-29.8751	1.0602E-13
TOT, (CKR+BOL), MEK	5	-42.5449	3.3342E-19
(TOT+CKR), (MEK+BOL)	6	-45.9113	1.1507E-20
TOT, (CKR+MEK+BOL)	7	-69.5026	6.5372E-31

The combinations of ecogeographical populations describing each hypothesis are shown. Bayes Factors are compared with that of the next-best model (including three identical runs for a four-population null model). Parentheses indicate populations merged. LBF, Log Bayes Factor; Pr(H), probability of the model (computed as marginal likelihood of model over the sum of the marginal likelihoods of all models compared).

As mentioned above, Migrate-N assumes constant population structure over time. Consequently, migration rates were also estimated using IMa2p and the metapopulation model chosen using Migrate-N. The CKS (TOT) taxon had to be excluded because runs with all three taxa (BOL, WBR and CKS) failed to converge within 48x(12x3.4GHz) hxCPUs during test runs or crashed after the burnin. Again this was possibly a consequence of the smaller sample and effective-population size of TOT relative to the lotic populations. TOT was not simply merged with WBR because TOT had appeared as a highly discrete population throughout the population structural analyses. Its merger with any other data partition was therefore unjustified and its exclusion saved computational time. No significant autocorrelation was found among any of the parameters estimated (suggesting that the data were sufficient to distinguish each of them), and ESS values for all exceeded 150. Table 6 shows that both population size parameters were consitently estimated (especially that for WBR) and imply effective population sizes of around 310000 (WBR) and 120000 (BOL). The greater size of WBR is expected as it includes 300 km of the lower Mekong river itself, whereas BOL is dominated by smaller highland streams. Although indicating the same relative proportions, the estimates of N_e_μ from Migrate-N (Table 4) are much lower; this may be explained by the use of a mutation rate prior (from BEAST) in the IMa2p analyses. As BEAST was focused on estimating mutation rate associated parameters, whereas Migrate-N estimated both mutation and migration rates, and population size, the mutation rate from BEAST was considered to be a better indicator of the true rate.

**Table 6:** Parameter estimates for a two-population model using IMa2p.

log P(D|G)	N_e_μ (BOL)	N_e_μ (MEK)	N_e_*M*_0_/2	N_e_*M*_1_/2	t_0_
-7563.3880	15.61 (11.3,20.3)	41.88 (40.7,42.5)	0.0698 (0.00#?,0.25 #?)	0.0817 (0.000#?,0.208#?)	15.54 (10.06,22.29)
-7581.2080	18.20 (11.4,26.9)	41.83 (40.5,42.5)	0.0339 (0.012#,0.049#)	0.0202 (0.018#,0.022#)	19.03 (9.66,24.99)
-7673.4100	17.14 (11.9,22.4)	41.88 (40.6,42.5)	0.0046 (0.000#,0.014#)	0.0037 (0.000#,0.007#)	12.76 (2.34,23.14)
	**16.06 (11.0,22.1)**	**41.88 (40.7,42.5)**	**0.0012 (0.000**#,**0.003**#**)**	**0.0096 (0.006**#,**0.013**#**)**	**15.98 (9.74,24.96)**

The results of three replicate runs are given, with estimates based on combined genealogies from all the runs in bold. Mean parameter estimates are followed by HPD in parentheses. *M* is migration rate per gene copy generation. N_e_*M*_0_ is twice the rate at which genes in BOL are replaced by those migrating in from WBR, and N_e_*M*_1_ is twice the rate in the opposite direction (BOL→ WBR). Parameter t_0_ is the splitting time estimate for the two populations in generations and in units of μ.

^?^, HPD estimates unreliable (interval appears discontiguous, e.g. >1 peak);

^#^, no upper and/or lower tail seen (truncated or flat HPD) so that HPD intervals will shift if the prior distribution is changed. The length of each run was as follows, by row (in steps per chain of 10 chains run simultaneously): 241300, 1229340, 1269023.

In contrast to population size, the migration rate estimates were less consistent (Table 6) and the HPDs appeared dependent on the prior (i.e. the HPD did not tail off toward zero at either the upper or lower limit of the prior). Nevertheless, the greater population migration rate is seen in the BOL→ WBR direction, which may be expected consdiring that rivers such as the Sre Pok and Xe Kong (of BOL) drain into the lower Mekong river (MEK of WBR). In contrast, much less variation was seen in estimates of splitting time. The analyses indicated that the BOL and WBR populations split 117793 (71797,183988) years prior to sampling. The mean TMRCA across all three main runs was quite consistent, being 43.22±1.16 (±SD); this approximates to 320000 generations ago.

Uninformative data can appear to describe an island model if a high upper bound is set as a prior on the splitting time of the ancestral population, as such analyses tend to infer a splitting time at that upper bound. To exclude this possibility, runs were performed with -t set to 25% of the value indicated by the test runs. Despite the reduction in the prior, the same high estimate of splitting time was returned; this suggested that the data were consistent with an island model.

### Bayesian inference of phylogeny and divergence times

**Fig 8:**
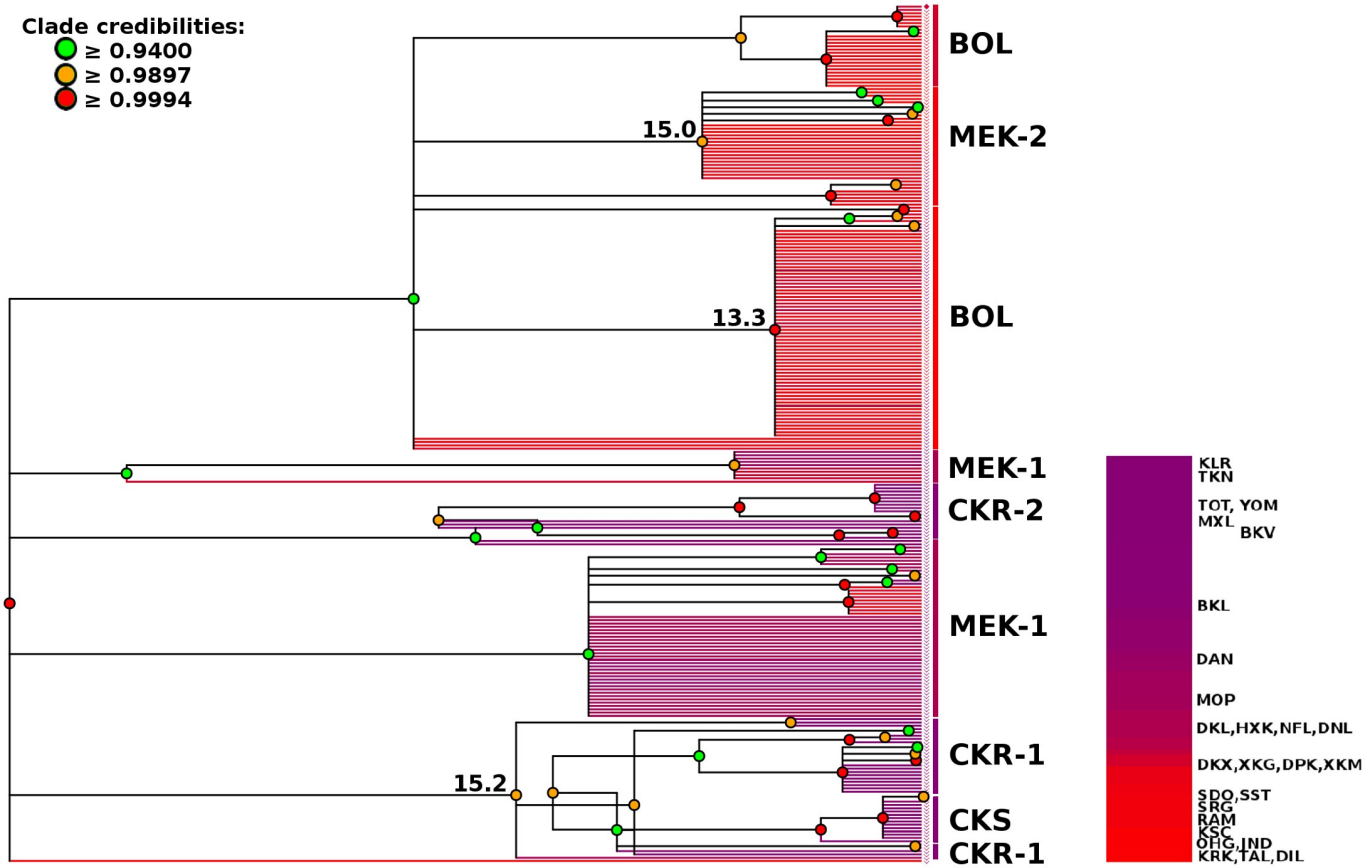
Majority rule (50%) consensus tree for *Neotricula γ-aperta*. To allow for phylogenetic uncertainty, this ultrametric tree is a summary of the MCC trees for three replicate runs of BEAST. Consequently, regions of topological incongruence among the runs are shown as unresolved (i.e. polytomies). Terminal branches are coloured according to the geographical position of the population from which the individual was sampled, relative to the northwestern most population (KLR). The legend shows the colour of each population. The clades are also labelled with the spatial-genetic cluster (identified by sPCA) to which their constituent sampling localities belong, and divergence time estimates are given for key cladogenic events (in Ma at the root of the clade).

Fig 8 shows the 50% majority rule consensus tree from phylogenetic estimation using BEAST. The tree depicts a phylogeny in which the four ecogeographical populations described in the previous sub-section, as a result of sPCA, did not appear monophyletic, except for CKS which formed an inclusive sub-clade within one of the two CKR clades; however, BOL and MEK were paraphyletic. BOL appears tripartite, divided into a large clade made up of snails from the Mekong-tributary rivers of northeastern Cambodia, a smaller clade of snails from the Attapeu region of southeastern Laos, and four monotypic clades (RAM1, RAM2, JND and DIL), all arising from a common ancestor with MEK-2 in an unresolved polytomy. MEK-2 comprised of the Mekong river taxa of the southerly limit of the range in Cambodia. MEK-2 showed three sub-clades whose relationships are unresolved (i.e. forming a trichotomy from a common ancestor with the six BOL clades). Interestingly, MEK-2 did not include any snails from the rivers of northeastern Cambodia that drain into the Mekong in this region (these clustered with some MEK-I (SDO) taxa and the larger BOL sub-clade).

The MEK-1, (CKR-1,(CKS,MXL)), CKR-2 clades form a basal polytomy. Within the CKR-1 clade, a trichotomy describes a monotypic clade (MOP), trifurcate clade (YOM), and a polytomic clade (six branches), within which CKS (TOT) forms a monophyletic clade and KLR and TKN appear respectively monophyletic on a bifurcate clade. The remaining four branches comprise of MXL and YOM. The two CKR clades differ in that CKR-1 includes CKS and TKN, and CKR-2 includes BKV taxa. The main clade of CKR-2 comprises a (BKV,(YOM)) clade, an (YOM,(MXL)) clade, three monotypic MXL clades, with two monotypic BKL clades basal to this main clade.

The standard deviation on the mean branch rate estimate for the uncorrelated relaxed exponential clock used was low (0.0019); this suggested that meaningful divergence times may be obtained by sampling the posterior. The marginal probability densities of the divergence time estimates showed HDIs (95% highest posterior probability densities) of around 4 Ma; their means are given in S2 Table. The mean divergence time priors (S1 Table) used fell below the lower limit of the HDIs for all estimates (suggesting that these distributions were not simply reflecting the priors). The HDIs of all three divergence time estimates showed partial overlap (~50%) and so could not be reliably ordered in time. Nevertheless, the CER divergence may have followed the CLM and NSP divergences; the latter being apparently isochronous at around 15 Ma. The divergence of the CER clade was estimated as having occurred around 13 Ma. The divergence time estimates are also given on Fig 8 at the root of the relevant clades. A per-lineage mean clock rate of 0.85% (substitutions/site/Ma) was estimated across the time depth of the phylogeny (see S2 Table).

## Discussion

### Population structure and geographical variation

The study found that population genetic variation corresponded with snail ecology or habitat. The fact that Ward’s clustering almost exactly recovered the eco-geographical structure of the samples, strongly suggests that *N*. *aperta* is not panmictic and that there is limited gene-flow among these regions. PCA indicated a marked clinal disjunction between the North and South of the range, with perhaps two, less well defined, sub-populations in the South (Fig 3). The disjunction was located between Khammouanne in Laos and sub-populations south of the northern boundary of the Bolavens plateau (including populations of Ubon, Northeast Thailand). Parzen plots of genetic distance *versus* ‘river distance’ suggested that the North-South divide was a consequence of well differentiated populations geographically far apart, rather than true IBD along extended clines. The contagious distribution indicated by the Moran scatter-plot for the Khammouanne spring-dwelling snails is consistent with their being endemic populations restricted to their host spring and possessing poor dispersal capabilities. The finding does not suggest that snails found in these springs were recently swept in from major-river populations during flood events. In contrast, snail populations occupying the central part of the range (e.g. the Mekong of southern Laos) appear far more panmictic. Such panmixis is most likely the result of mixing among sub-populations during the annual flood, and the ready interconnection of the rivers involved. Nevertheless, these populations still showed significantly positive spatial auto-correlation, which implies that each snail is more likely to be surrounded by individuals more closely related to it than would be expected if the distribution were random (i.e. spatial-genetic affinity in the snails showed granularity). Snails of the most southeasterly populations (i.e. the rivers of northeastern Cambodia) appeared to be surrounded by individuals less closely related to them than expected in the absence of population structure. This may be due to a lack of migration among the rivers of this region despite their geographical proximity, which leads to the existence of spatially close but genetically distant individuals.

sPCA and data regression onto MEMs indicated highly significant global population structure, but much less local structure. The North-South divide suggested by PCA was reinforced by plotting of lagged principal scores onto geographical space. The existence of a third sub-population comprising the sampling sites of Northeast Cambodia and the Xe Kong drainage of Laos (effectively the Bolovens sub-population, BOL) was supported by the second PC of the sPCA, and to a lesser extent by the minimum variance clustering dendogram for the first PC (of the sPCA), where it appears as a sub-clade of the major southern clade (Fig 6B).

Ward’s clustering identified four ecogeographical populations whose membership corresponded almost directly with the snails sampled within the geographical region of each of the four inferred clusters. Such a remarkable correspondence raises the possibility that ecogeographical differences sub-divide *N*. *aperta* and that some degree of ecological, as well as physical, isolation might be present. The sub-populations inferred by sPCA were also consistent with these ecogeographical clusters (Figs 1 and 6).

### Phylogenetics and dating

In contrast to Ward’s clustering, phylogenetic estimation using BEAST, found only CKS to be monophyletic (and only as a sub-clade of one of two CKR clades). It should be noted, however, that whilst PCA and related methods are based solely on genetic differences, BEAST attempts to recover history and could give a better estimate of the ancestral relationships of the sub-populations. The BEAST phylogeny (Fig 8) suggests two Mekong river clades, one of southern Laos (MEK-1) and the other of Cambodia (MEK-2). Although the Cambodian Mekong river clade did not contain any of the taxa from populations of the Northeast Cambodian rivers that drain into the Cambodian Mekong, it did appear to share a common ancestor with taxa from these rivers and from the Attapeu region of Laos in the North (i.e. with the two Bolovens clades). Nested clade analysis [79] previously published for an earlier (more limited) data-set [16] also suggested a disjunction between Lao and Cambodian Mekong river populations, with the latter having closer affinity with a Bolovens clade. Although the two stretches of the Mekong river are separated by the cataract at Khone falls, this is unlikely to represent a significant barrier to downstream dispersal. Consequently, either historical or ecological factors may explain the relatively deep evolutionary divergence between MEK-2 (and BOL) from all other taxa. Average levels of dissolved oxygen (2002–2014) were reportedly higher in the MEK-2 stretch of the Mekong river [80], and it could be that MEK-1 populations have acquired adaptations to lower oxygen levels (or associated habitat differences) such that they are disadvantaged when migrating southwards into MEK-2 habitats. In terms of phylogenetics, the Red river hypothesis [81] proposes a Pliocene colonisation of Laos, by proto-*Neotricula* from Hunan, via Cambodia, with snails radiating northwards into Laos from northeastern Cambodia. The two hypotheses are not mutually exclusive and are difficult to distinguish using the present data; however, the use of rapidly evolving markers such as microsatellites may shed light on the processes involved (by emphasising the effects of very recent or ongoing (i.e. ecological) events).

Confidence intervals for divergence time estimates (from BEAST) were wide, such that it is not possible to order the times of the MRCAs of all three major clades in Fig 8. Nevertheless, the common ancestor of the Bolovens clade may be more recent, at around 13 Ma, than those of MEK-2 (CLM) and CKS+CKR (NSP), which appear isochronous at just over 15 Ma. These values are much greater than the estimated TMRCA for all of the taxa (excluding TOT) from IMa2p, which was around 0.3 Ma. Similarly, the splitting time between BOL and WBR was estimated at just over 0.1 Ma. It should be noted that whilst IMa2p estimated splitting times between contemporary populations, BEAST estimated dates for divergence events among ancestral populations. Further, the TMRCA can be much older than the most basal node of the phylogeny, which in the IMa2p analysis is that representing the ancestor of BOL and WBR if the depth of the phylogeny is less than 4-7N_e_ [82] (here this is around 10x greater at 1.7–3 Ma).

As in the 2008 study (based on the *rrnL* data only and fewer sampling sites), the order of divergence events appears to be near simultaneous divergence of the spring-dwelling Cammon karst and Cammon karst stream clades (NSP), and of MEK-2, followed by that of BOL. In contrast, Attwood et al. (2008) [16] dated these events at 10, 10, and 6.5 Ma rather than the 15.2, 15 and 13.3 inferred here. Nevertheless, the relative intervals are similar as are the absolute values when the wide confidence intervals are considered. In contrast to the earlier study, good congruence was observed between ecogeographical populations and genetic variation, whereas the 2008 paper did not infer a monophyletic BOL clade, instead BOL taxa were divided between MEK-1 and MEK-2. The pectinate appearance of the major clades in Fig 8 suggests rapid radiation of the snails soon after arrival of the founding population in each region (MEK-1, MEK-2, BOL, etc). This is in keeping with the idea that the Southeast Asian triculinae entered the region via the exposed Sunda shelf (now off the Vietnam coast) and founded the Cammon karst spring populations; thus retaining the same habitat as ancestral Triculinae in Hunan [73]. The founding population may have diverged into riverine forms and colonised Khorat basin, Bolovens plateau and Cambodia just prior to the uplift of the Bolovens plateau, around 15 Ma [83], and major isolating orogenic events.

### Migration among regions and population size

Values of the population migration rate (Table 6) were much less than the threshold value of 1, at which point gene-flow begins to create the appearance of a single population [84]; therefore it was extremely unlikely that *N*. *aperta* exists as a single panmictic population. Nevertheless, unexpectedly high levels of migration were observed between CKS (TOT) and BOL and WBR (i.e. MEK+CKR); these were attributed to systematic error caused by the relatively small sample size and underestimated θ of CKS. In support of this, Attwood et al. (2008) [16], who also used Migrate (but not also IMa2p), and did not cluster CKS apart from CKR, reported values for N_e_*M* ranging from 0.000 to 0.005 between CKS+CKR and BOL+MEK1 or BOL+MEK2. Exclusion of CKS, allowed estimation of N_e_*M* between BOL and WBR which was found to be close to zero (0.001) WBR→BOL and almost ten times higher BOL→WBR; this is expected as *N*. *aperta* is thought to show very poor survival out of water. Even dispersal downstream (BOL→WBR) is likely to occur at low rates because habitats suitable for this snail are highly discontinuous [9]. The higher rate BOL→WBR is consistent with some colonisation of the Cambodian Mekong by snails from the rivers of Northeast Cambodia.

Estimates of N_e_ between 81084 and 313281 are very high. Even for dense stands of gregarious molluscs such as *Ostrea edulis* (the European flat oyster) N_e_ is reportedly only around 23000 [85]. *N*. *aperta* is much smaller than the aforementioned oyster, and lithic substrata provide a vast surface area along their ridges and crevices; therefore *N*. *aperta* populations may be very large. Although there is often no linear relationship between snail population density (or even density of infective snails) and prevalence of schistosomiasis in humans, the existence of very large numbers of snails around human settlements, each snail shedding few cercariae, is likely to favour transmission of *S*. *mekongi*. It is worth noting in this context, that in comparison with *Biomphalaria glabrata* (Say 1818) transmitting *Schistosoma mansoni* Sambon 1907, where one snail may shed over 2000 cercariae per day [86] (and prevalence of infection in the populations can be over 75% [87]), the cercarial ouput of *N*. *aperta* is much lower (as few as 23 per day per snail, with prevalence of 0.22% [88]). Most of the areas predicted to harbour *N*. *aperta* are inaccessible. The habitats that have been surveyed display extremely high population densities; for example, densities greater than 5000 snails per m^2^ have been reported for *N*. *aperta* in the Xe Kong river [89] (note this was prior to completion of the Nam Theun 2 dam). The present observations suggest that such high densities may be a feature of *N*. *aperta* populations across most of its range, including unsurveyed reaches of the upper rivers.

### Conclusions

The findings of the present study were not inconsistent with a colonisation of Laos and Cambodia via southern Vietnam and into the Bolovens region; however, an earlier date (Miocene) was estimated relative to that reported by Attwood et al. (2008) [16]. In this respect the present date estimates agree with those of earlier phylogenetic studies [13,14]. The observation that, what little gene-flow there is in *N*. *aperta* was westwards from Bolovens to the other regions offers some support to a Sunda shelf-Vietnam colonisation; however, this pattern of gene-flow is relatively recent, with phylogenetics suggesting rapid colonisation of the entire range from the Cammon region and into Bolovens, just prior to isolating events associated with uplift of the Bolovens plateau (*c*.*a*. 15 Ma). Consequently, the results imply that the present distribution of *N*. *aperta* is more a result of history than of ecology, and that this snail is not currently necessarily currently limited by the distribution of suitable habitats. Nevertheless, dispersal capabilities appear to be very low (for example, closely parallel river courses in Cambodia harbour divergent populations) and expansion of the range is likely to occur at very low rates, even with human activity. Hydropower expansion, with 133 dams completed or proposed for the lower Mekong basin [90], is likely to effect gradual changes the distribution of snails, as changes in water depth create new suitable habitats [9]. The results confirm that the radiation of these snails and their associated schistosomes was heterochronous; this has implications for understanding of the snail-parasite association. Heterochronous evolution gives less opportunity for co-evolutionary arms races as proposed for other human schistosomes [91]. In this sense the findings support the conclusions of studies on *S*. *japonicum*, that ecological factors (e.g. schistosomes evolve to avoid snails that would release their cercariae in habitats that do not favour transmission) are perhaps more important than phylogenetics, and that host switches are more likely than previously thought [92]. The likelihood of host-switching in *S*. *mekongi* and other Asian species, relates to the chance of the parasite escaping the effects of snail control efforts and requires further investigation.

The present study supports view that the spring-dwelling *N*. *aperta* of the Cammon plateau in Laos are distinct ecotype, and that there is a marked lack of gene-flow between the northern and southern halves of the snail’s range. Consequently, further investigation is required to assess the potential for spring-dwelling snails to act as intermediate hosts for *S*. *mekongi* and to support transmission in habitats currently assumed to be free of schistosomiasis. The remarkable correspondence between ecogeographical area and snail population genetic structure suggests that some degree of ecological adaptation, in addition to physical barriers, inhibits introgression between these regions. In turn this suggests that persistence of snail populations in the Mekong river through spate periods is not achieved primarily by colonisation from seeder populations in tributary highland streams that experience a less severe flood cycle, which has been proposed as an explanation for snail persistence [93]. The finding suggests that snail control in snail habitats in the upper reaches of tributaries will have little impact on persistence of schistosomiasis transmission in the Mekong river itself. The only exception might be the Bolovens populations, as there is some gene-flow apparent between those and Mekong river snail foci. The observation of fine-scale clustering (granular spatial-genetic associations) in the snail populations also has implications for disease control. The finding Implies that snail-mediated reintroduction of schistosomiasis, from outside of local snail or parasite control intervention areas, is unlikely because snail populations are made up of many small and relatively discrete micro-populations.

In summary, correspondence between ecogeographical sub-populations and clades identified phylogenetically, and by genetic distance based clustering, in the present study, illustrates the value of improved sampling (both geographical and genomic). The study has shown that *N*. *aperta* exists as a metapopulation at multiple scales, including down to a micro-population-level granularity; this has implications for the design of schistosomiasis control interventions. Similarly, the lack of gene-flow between tributary populations (except perhaps those of Bolovens) and those of the Mekong implies that the effects of Mekong river and highland stream interventions will be independent. Interpretation of phylogenetic reconstructions implies that history shapes the current distribution of *N*. *aperta* and some expansion of the range is possible, especially after hydropower development alters the regional hydrology. The study has confirmed limited gene-flow between Cammon plateau populations in the North of the range and other populations, and that spring-dwelling snails are probably a distinct ecotype. Further work is needed to assess the epidemiological significance of the spring-dwelling taxa. The extent of the analyses was limited by the number of characters sampled and future studies should use more extensive sampling of the genome, which is now becoming more practical as technology improves. The present work used pre-existing data that included some very remote and/or now extinct sub-populations, as well as newly published data. The work therefore provides a record of the population-genetics of *N*. *aperta* prior to the impact of extensive hydropower development in the region, as well as an indication of the potential for range expansion as well as predictions of responses to schistosomiasis control.

### Ethics statement

Field Research: Material from Laos was collected with the permission of the Ministry of Public Health Lao PDR. Material from Cambodia, with the permission of the Cambodia National Malaria Center. Material from Thailand, with the permission of Mahidol University (Faculty of Science).

## Supporting information

S1 Table Settings for BEAST runs used in phylogenetic estimation and divergence dating. Exponential distributions are described as mean, offset.
(CSV)

S2 Table Results of a Bayesian estimation of divergence times and nucleotide substitution rates for replicate runs. The estimates are mean ± SE (Standard Error of the mean). ESS, Effective Sample Size (i.e., size corrected for auto-correlation); HDI, the 95% highest posterior probability density. The meanRate is the average rate for the branches on the tree weighted by branch length and expressed in substitutions/site/Ma.
(CSV)

S1 Fig Plot of log marginal likelihood against chain length for different length runs of BEAST with optimal settings. (PNG)

S2 Fig Simulated and observed values for a mantel test comparing Edwards’ genetic distances and river distances for individual *Neotricula γ-aperta* snails. The observed value is indicated by the black diamond symbol.
(PNG)

S3 Fig Local (Parzen) 2-dimensional density plot for genetic distance and ‘river distance’ for individual *Neotricula γ-aperta* snails. (PNG)

S4 Fig Results of a PCA for genetic structure within *Neotricula γ-aperta*. A, Parzen scatterplot for distribution of genotypes along the first two PCs. B, a loading plot for the PCA.
(PNG)

S5 Fig Density plot of Moran’s I values in Monte-Carlo sampling. Plots are shown for the first (A) and second (B) PCs in a PCA of standardised allele frequency data for *Neotricula γ-aperta*.
(PNG)

S6 Fig Results of Monte-Carlo simulations and observed value for a Mantel test of correlation between scaled-genetic and spatial distance for *Neotricula γ-aperta*. The observed value is indicated by the black diamond symbol.
(PNG)

S7 Fig Eigenvalues for an sPCA of *Neotricula γ-aperta*. A, scree-plot of composite eigenvalues, with first two PCs in red. B, Decomposition of sPCA into Moran’s I against variance for each PC.
(PNG)
