# Supplementary figures and images for: Population genetic structure and geographical variation in Neotricula aperta (Gastropoda: Pomatiopsidae), the snail intermediate host of Schistosoma mekongi (Digenea: Schistosomatidae)
Source: PLoS Negl Trop Dis. 2019 Jan 28;13(1):e0007061. doi: 10.1371/journal.pntd.0007061 (PMC6366693; doi:10.1371/journal.pntd.0007061)

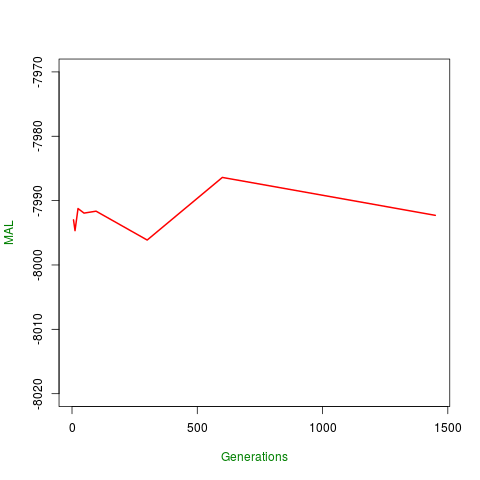

Supplement: S1 Fig — (PNG) [file pntd.0007061.s003.png]

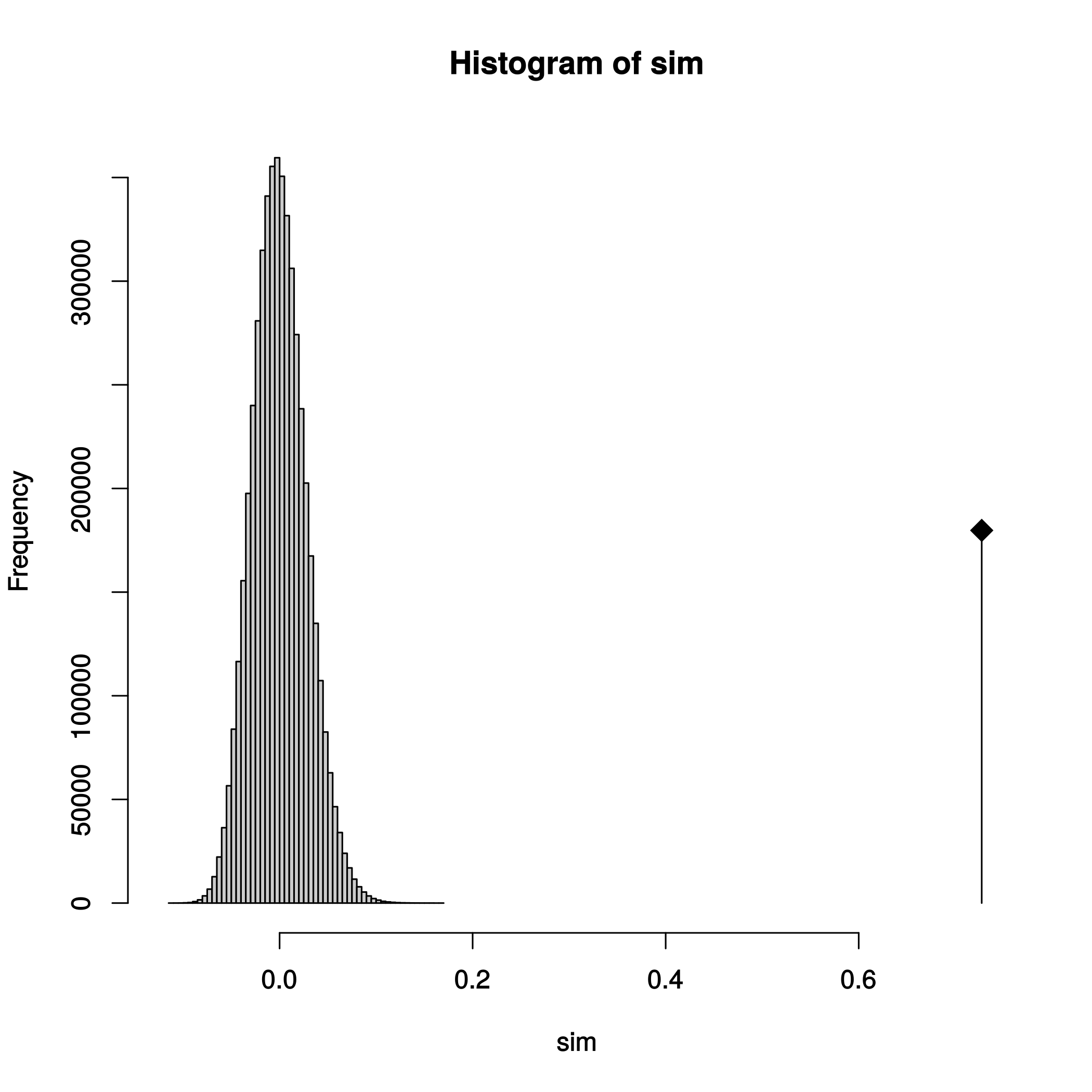

Supplement: S2 Fig — The observed value is indicated by the black diamond symbol. (PNG) [file pntd.0007061.s004.png]

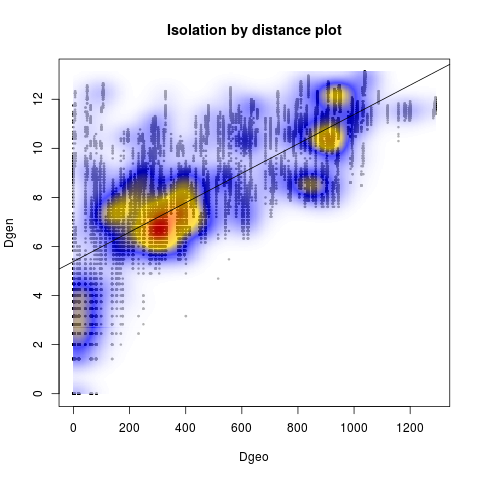

Supplement: S3 Fig — (PNG) [file pntd.0007061.s005.png]

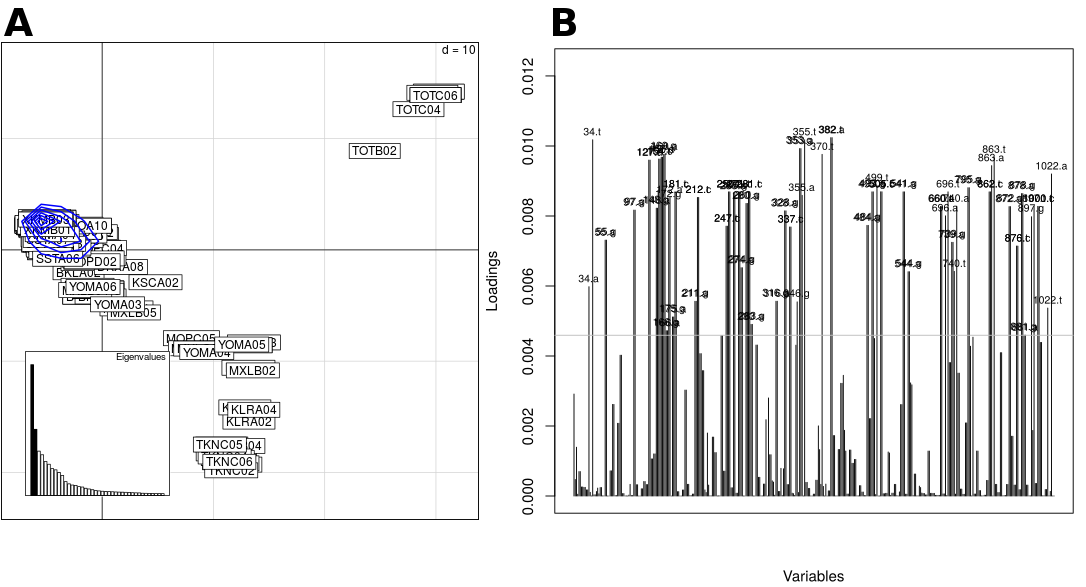

Supplement: S4 Fig — A, Parzen scatterplot for distribution of genotypes along the first two PCs. B, a loading plot for the PCA. (PNG) [file pntd.0007061.s006.png]

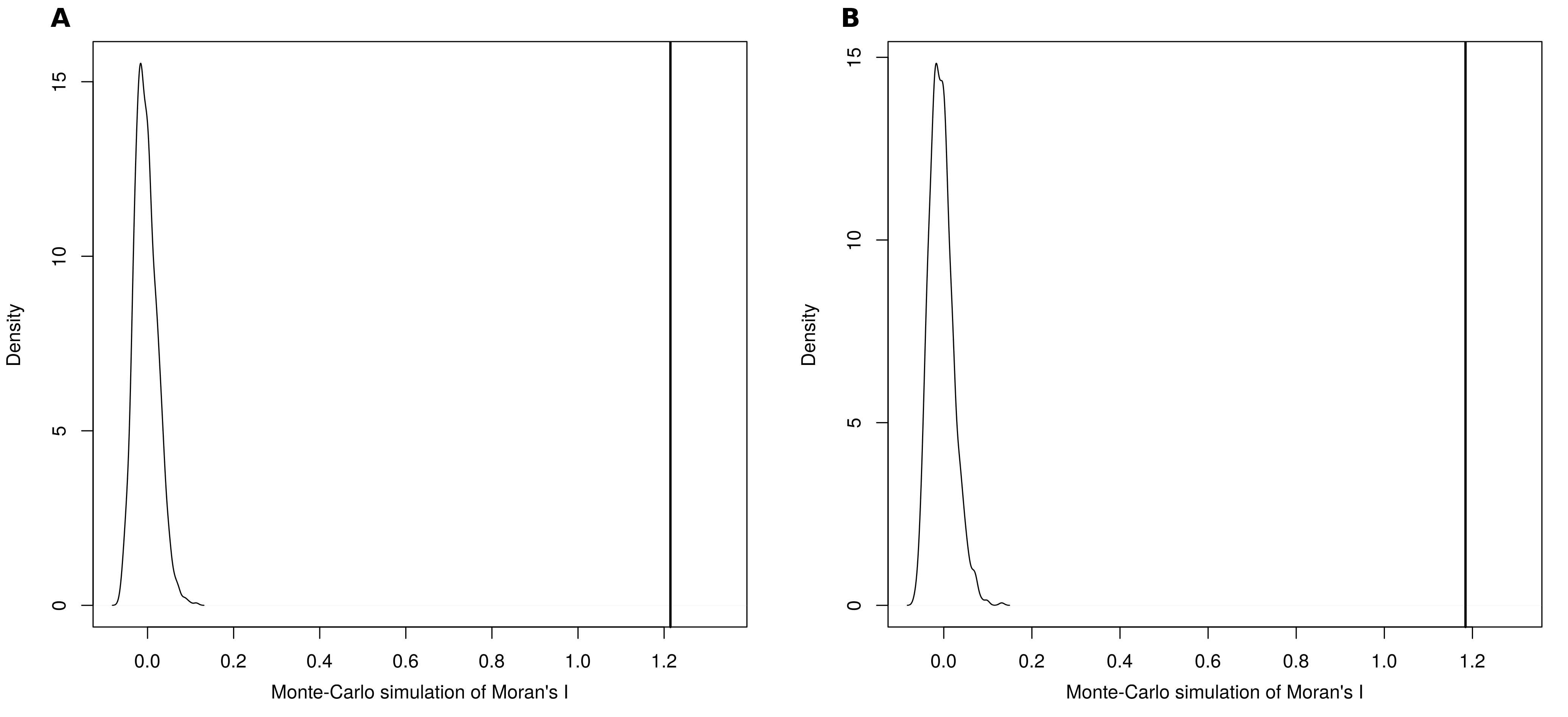

Supplement: S5 Fig — Plots are shown for the first (A) and second (B) PCs in a PCA of standardised allele frequency data for Neotricula γ-aperta. (PNG) [file pntd.0007061.s007.png]

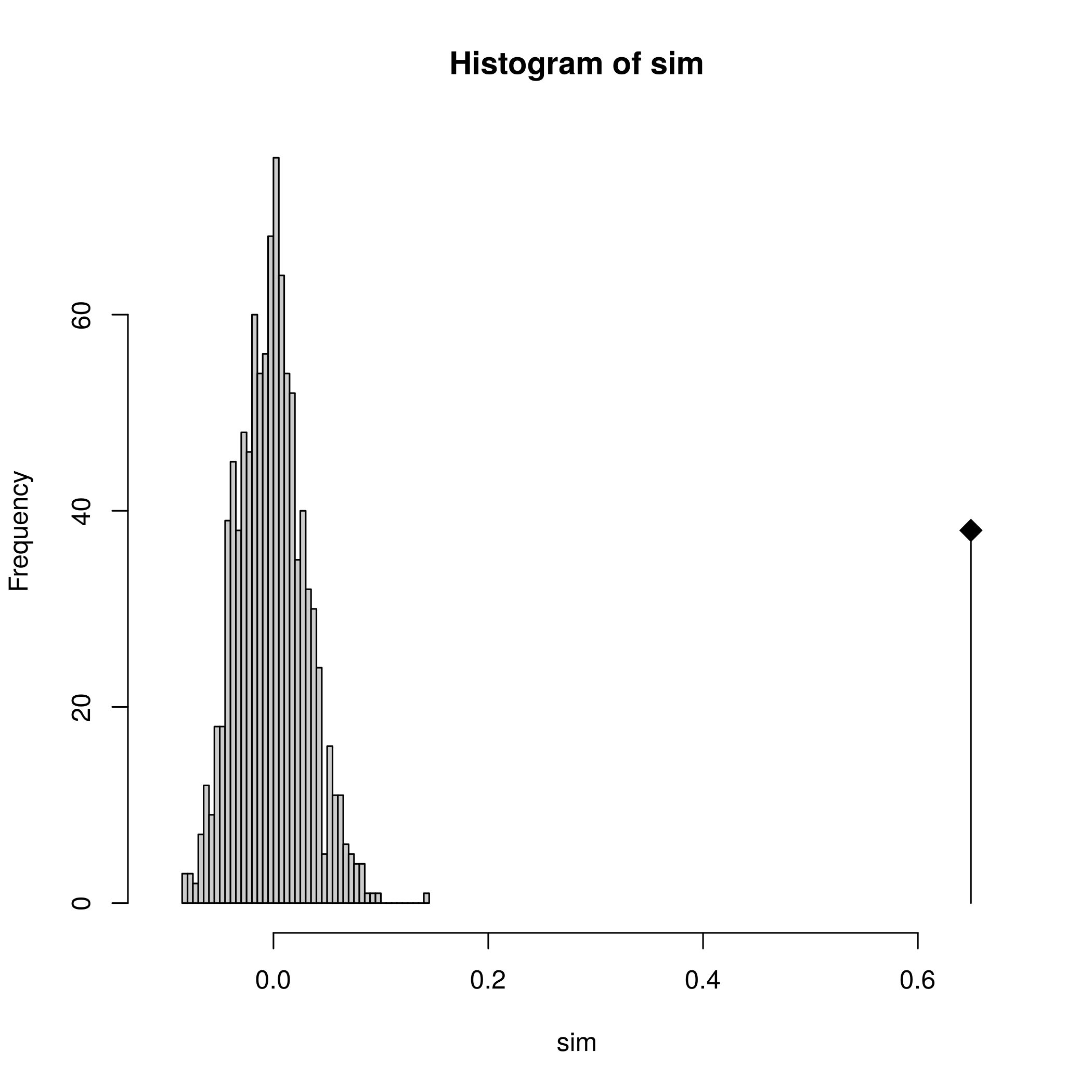

Supplement: S6 Fig — The observed value is indicated by the black diamond symbol. (PNG) [file pntd.0007061.s008.png]

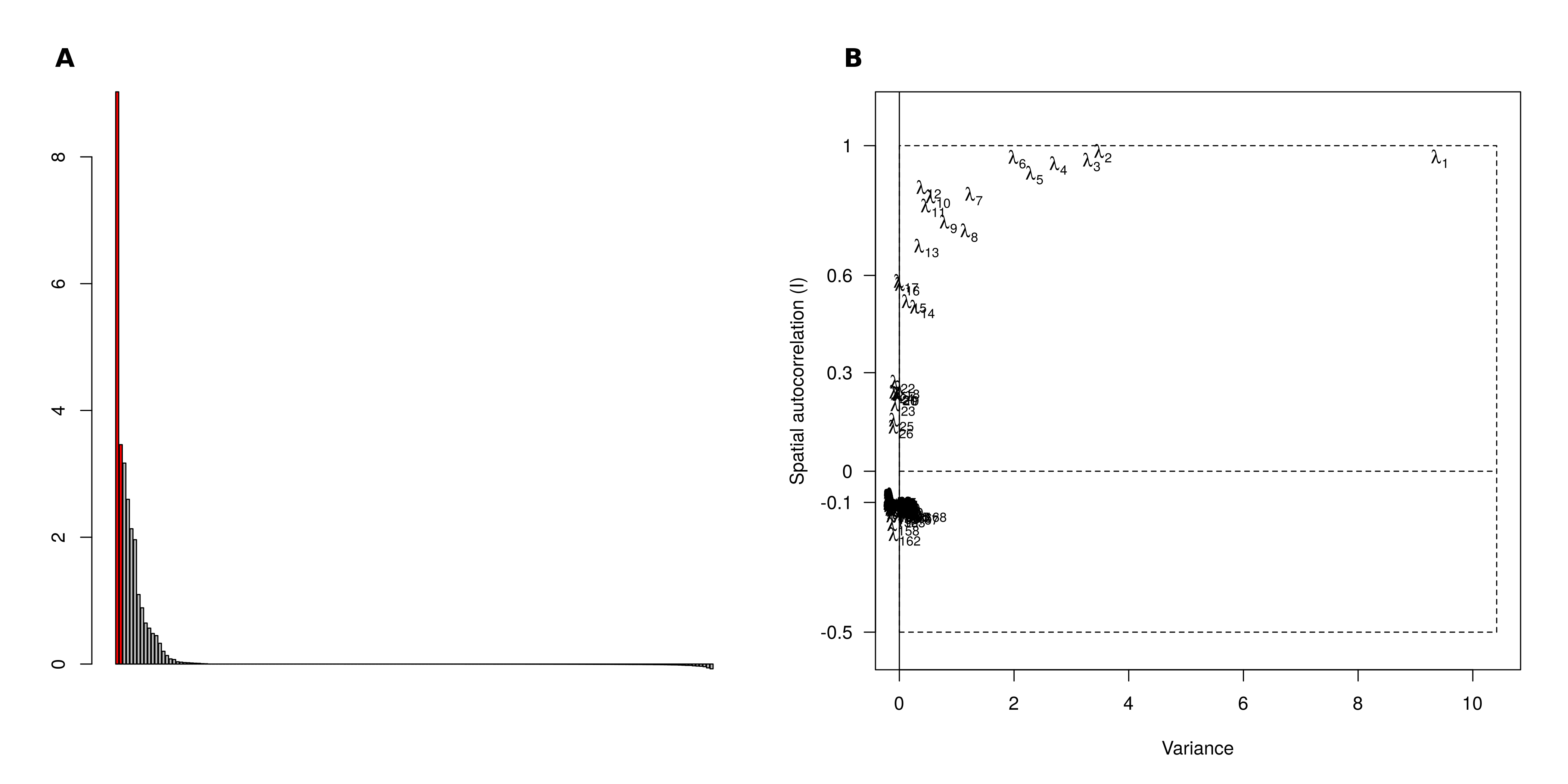

Supplement: S7 Fig — A, scree-plot of composite eigenvalues, with first two PCs in red. B, Decomposition of sPCA into Moran’s I against variance for each PC. (PNG) [file pntd.0007061.s009.png]
